# Chemical Composition Analysis and Hypolipidemic Activity of Polyphenols Isolated from Small-Leaved Kuding Tea

**DOI:** 10.3390/foods15203667

**Published:** 2026-10-15

**Authors:** Yingying Xiao, Zhoujie Yang, Huaiqiang Zhang, Yan Xue, Yunjie Feng, Bing Lin, Hui Shi, Ying Zhou, Tingting Feng

**Affiliations:** 1 School of Pharmacy, Guizhou University of Traditional Chinese Medicine, 4 Dongqing Road, Guiyang 550025, China; 2 Guizhou Key Laboratory of Modern Traditional Chinese Medicine Creation, Guiyang 550025, China; 3 Research Center for Application and Development of Medicine and Food Dual-Use Resources, Guizhou University of Traditional Chinese Medicine, Guiyang 550025, China

**Keywords:** small-leaved kuding tea, polyphenols, hyperlipidemia, target

## Abstract

Hyperlipidemia is a widespread metabolic disease that triggers abnormal lipid accumulation. Plant polyphenols possess great potential for lipid metabolism regulation. Small-leaved kuding tea (*Ligustrum robustum* Roxb. Blume, SLKDT) is abundant in polyphenols with proven hypolipidemic activity, whereas its key active constituents and molecular targets remain unclear. This study aimed to identify the key active components and core targets responsible for the lipid-lowering effects of SLKDT polyphenols. Response surface methodology (RSM) was employed to optimize the extraction process of SLKDT polyphenols. In vitro cell experiments were performed to verify the lipid-lowering effect of SLKDT polyphenols, while the obtained polyphenols were further qualitatively characterized via LC-MS/MS. Furthermore, network pharmacology prediction combined with molecular docking was used to screen the key bioactive compounds and their target proteins associated with lipid metabolism regulation. Under the optimal extraction conditions (43% ethanol concentration, ultrasound time of 40 min, solid-to-liquid ratio of 1:56 g/mL, and ultrasonic temperature of 57 °C), the yield of polyphenols from SLKDT was 131.88 mg/g. In vitro experiments confirmed that SLKDT polyphenols significantly reduced intracellular lipid accumulation in HepG2 cells, increased the activities of antioxidant enzymes including GSH-PX, CAT, and T-SOD, and decreased MDA content. Furthermore, SLKDT polyphenols markedly downregulated the expression of lipid synthesis-related genes, including *ACC*, *HMGCR*, *SREBP-1c*, and *FASN*, while upregulating *CYP7A1* expression. A total of 88 compounds were Preliminary identified by LC-MS/MS, mainly including glycosides, flavonoids and phenolic acids. Network pharmacology analysis revealed that chrysin, 6-demethoxytangeretin, bavachinin, isobavachin, and bavachalcone were the core components mediating the lipid-lowering activity of SLKDT polyphenols, with target proteins including *TP53*, *STAT3*, *AKT1*, *HSP90AA1*, and *EGFR*. This study elucidates the key components and critical targets underlying the lipid-lowering effects of SLKDT polyphenols, providing experimental evidence and theoretical support for the development of SLKDT-derived products and natural lipid-lowering agents.

## 1. Introduction

Hyperlipidemia is defined as abnormally elevated serum lipid concentrations caused by disorders of lipid metabolism or transport [1]. As one of the most common lifestyle-related diseases, hyperlipidemia not only promotes excessive lipid accumulation in arterial walls to induce vascular occlusion, but also impairs endothelial structural and functional integrity, thereby leading to cardiovascular and cerebrovascular diseases (CVDs) [2,3]. It has evolved into a critical public health concern worldwide, whose global incidence and mortality surpass those of infectious diseases and malignancies. Epidemiological data indicate that approximately 40% of Chinese adults present hyperlipidemia of varying degrees, and the condition features a younger-onset trend, which poses a critical public health challenge in China [4,5]. Despite the favorable therapeutic efficacy of statins and fibrates for clinical management of hyperlipidemia, long-term administration of these agents may induce a spectrum of adverse side effects, such as gastrointestinal disorders, hepatorenal toxicity, and myopathy [6]. Hence, developing natural bioactive agents to regulate lipid homeostasis with excellent safety and long-term applicability represents a trending research focus in functional food and pharmaceutical fields.

As ubiquitous plant secondary metabolites, polyphenols mainly consist of flavonoids, phenolic acids, tannins, anthocyanidins and lignans, which are widely enriched in fruits, vegetables, nuts, tea, cocoa, red wine and multiple cereals [7,8,9]. Polyphenols are extensively applied as functional dietary ingredients in functional foods and nutritional supplements due to their potent antioxidant, anti-inflammatory and metabolic-modulatory capacities, and are defined as the seventh essential nutrient for humans [10]. Currently, polyphenols have been validated to exert multi-target lipid-regulating effects by inhibiting de novo adipogenesis, restraining intestinal cholesterol uptake, and mitigating vascular oxidative inflammation [11]. Mechanistic studies indicate that polyphenols stimulate the *AMPK* signaling pathway. They inhibit lipogenic transcription factors *SREBP-1c* and *FAS* to lower hepatic and adipose lipid buildup, and enhance *PPARα*-mediated fatty acid β-oxidation [12]. Additionally, polyphenols can also inhibit the activity of intestinal pancreatic lipase, attenuate dietary lipid uptake, reduce serum total cholesterol (TC), triglyceride (TG) and low-density lipoprotein cholesterol (LDL-C), high-density lipoprotein cholesterol (HDL-C) concentrations, and alleviate hyperlipidemia and obesity [13]. Although polyphenols are widely acknowledged for their lipid-regulating capacities, significant differences in hypolipidemic activities exist among polyphenol subtypes derived from different plant sources. Of all plant-originated polyphenols, tea polyphenols are the most extensively explored edible subgroup for preventing and adjuvantly treating metabolic syndromes. As the primary bioactive components of tea, tea polyphenols also determine the tea’s color, aroma, and flavor [14].

SLKDT is an endemic tea plant native to Southwest China. It has a documented history of nearly 2000 years as a folk herbal tea in China, and was officially approved as a conventional edible material by the National Health Commission of the People’s Republic of China in 2011 [15]. Studies have demonstrated that SLKDT exerts antioxidant, anti-aging, anti-inflammatory, lipid-regulatory and hepatoprotective capacities, as well as hypoglycemic effects [16,17,18]. Long-term SLKDT intake ameliorates hypertension and reduces the risk of cardiovascular diseases. Recent in vivo investigations further demonstrated that SLKDT extract alleviated high-fat diet-induced obesity and hepatic steatosis by modulating *PPARα/CPT1A*-mediated fatty acid oxidation and suppressing hepatic adipogenic transcription programs [19]. Previous studies have found that SLKDT extracts can alleviate pancreatic islet cell damage and elevate insulin secretion by modulating proteins associated with the *PI3K/AKT* pathway responsible for glycolipid metabolism and oxidative stress, thereby ameliorating pathological symptoms in T2DM mice [17]. Glycosides isolated from SLKDT have been validated to exert potent inhibitory activities against α-glucosidase and α-amylase [20,21]. In addition, polysaccharides derived from SLKDT exert potent antioxidant, anti-tumor and hypoglycemic bioactivities [16]. Although multiple bioactive fractions of SLKDT, including glycosides and polysaccharides, have been well characterized, most lipid-related mechanistic research was performed using crude SLKDT extracts. To date, limited information is currently available regarding the lipid-modulating mechanisms of polyphenols extracted from SLKDT, especially the systematic identification of polyphenol composition and the multi-target regulatory network underlying their lipid-lowering effects.

Therefore, this study aimed to further explore the regulatory effects and underlying mechanisms of SLKDT polyphenols on lipid metabolism. In this work, the optimal extraction procedure of SLKDT polyphenols was firstly determined via response surface methodology (RSM), and a high-lipid HepG2 cell model was constructed to validate the lipid-modulatory capacity of SLKDT polyphenols. Subsequently, the chemical constituents of SLKDT polyphenols were characterized by LC-MS/MS, and integrated network pharmacology and molecular docking were adopted to screen and verify the core targets implicated in the lipid-regulatory effect of SLKDT polyphenols. Finally, these findings of this study can provide a novel theoretical basis for the development of SLKDT polyphenol-based functional lipid-lowering dietary supplements and adjuvant therapeutic preparations.

## 2. Materials and Methods

### 2.1. Chemicals and Reagents

The SLKDT was purchased in Yuqing County, Guizhou Province. Pancreatic lipase and cholesterol esterase were purchased from Shanghai Yuanye Biotechnology Co., LTD. (Shanghai, China). Palmitic acid BSA high-fat cell additive was purchased from Meilun Bio Co., LTD. (Dalian, China). Human hepatoma cell HepG2, fetal bovine serum, DMEM high-glucose medium, trypsin-EDTA digestion solution, penicillin-streptomycin solution, and cell cryopreservation solution were purchased from TransGen Biotech Co., LTD. (Beijing, China). Orlistat, lauric acid-4-nitrophenyl ester and simvastatin were purchased from Shanghai Maclean Biochemical Technology Co., LTD. (Shanghai, China). Oil Red O staining solution was purchased from Biyuntian Biotechnology Co., LTD. (Shanghai, China). Lipase (LPS) test assay kit (Cat. No.: ADS-W-ZF005) was purchased from Jiangsu Edison Biotechnology Co., LTD. (Yancheng, China). Total protein quantification assay kit, T-CHO assay kit, TG assay kit, HDL-C assay kit, LDL-C assay kit, GSH-PX assay kit, MDA assay kit, CAT assay kit, T-SOD assay kit, MTT Cell Proliferation and cytotoxicity assay kit were purchased from Nanjing Jianxian Bioengineering Institute (Nanjing, China).

### 2.2. Sample Preparation

In total, 50.00 g of SLKDT powder was weighed and mixed with 43% ethanol at a solid-to-liquid ratio of 1:56 (g/mL). Ultrasonic-assisted extraction was carried out using an ultrasonic processor (XM-400UVF, Kunshan Xiaomei Ultrasonic Instrument Co., Ltd., Kunshan, China) at 57 °C, 400 W and 50 Hz for 40 min, and the extraction procedure was repeated three times. Afterwards, the mixture was centrifuged at 8000 r/min at room temperature with a TG16G centrifuge (Hunan Kaida Science and Technology Co., Ltd., Changsha, China). The supernatant was collected, concentrated, and lyophilized using a freeze dryer (Xinyi-10, Ningbo Xinyi Ultrasonic Equipment Co., Ltd., Ningbo, China) to obtain SLKDT polyphenol extract powder. The powder was stored at −20 °C in the dark for subsequent experiments.

### 2.3. Optimization of Polyphenol Extraction Process

To explore the key factors affecting the extraction yield of SLKDT polyphenols, single-factor experiments were conducted to investigate four extraction variables, including solid-to-liquid ratio (1:30, 1:40, 1:50, 1:60, 1:70 g/mL), ethanol volume fraction (20%, 30%, 40%, 50%, 60%), ultrasound time (20, 30, 40, 50, 60 min) and ultrasonic temperature (35, 45, 55, 65, 75 °C). On the basis of single-factor experimental results, response surface methodology (RSM) was performed via Design-Expert 12 software. Taking the polyphenol extraction yield as the evaluation index, the above four variables were set as independent experimental factors with three gradient levels for each factor. A total of 29 experimental runs were designed to optimize the ultrasonic-assisted extraction conditions of SLKDT polyphenols. The experimental factors and their corresponding levels are presented in Table 1.

### 2.4. In Vitro Lipid-Lowering Activity Assay

#### 2.4.1. Pancreatic Lipase-Inhibitory Activity Assay

The pancreatic lipase in vitro inhibitory assay was performed according to a previously described method [22]. 4-Nitrophenyl laurate was dissolved in absolute ethanol to prepare an 8 mmol/L substrate working solution. Pancreatic lipase powder was dissolved in PBS buffer to prepare a 10 mg/mL enzyme stock solution, which was diluted 10-fold with PBS buffer before experimentation. Briefly, 100 μL of polyphenol sample solution was mixed thoroughly with 200 μL of diluted lipase solution, followed by the addition of 200 μL 4-nitrophenyl laurate substrate solution. The resulting mixture was incubated at 37 °C for 20 min, and the absorbance value was measured at 405 nm immediately after incubation. The lipase-inhibitory rate of each polyphenol sample (1, 3 and 5 mg/mL) was calculated using the recorded absorbance data. Orlistat was applied as the positive control, and its absorbance was detected under the same experimental conditions.

Inhibition rate = [1 − (A − B)/(C − D)] × 100%

(A) Sample solution mixed with pancreatic lipase solution. (B) Sample solution mixed with PBS buffer. (C) PBS buffer mixed with pancreatic lipase solution. (D) Pure PBS buffer.

#### 2.4.2. Determination of Cholesterol Esterase Inhibitory Activity

The cholesterol esterase in vitro inhibitory assay was conducted following a reported protocol [23]. Briefly, 250 μL of phosphate buffer (0.1 mol/L) was added to the reaction system firstly, followed by the successive addition of 100 μL cholesterol esterase solution (0.1 U/mL) and polyphenol sample solution (1, 3 and 5 mg/mL). Afterwards, 100 μL of 4-nitrophenyl laurate solution (8 mmol/L) was supplemented into the mixture, which was incubated at 37 °C for 30 min. The absorbance of each mixture was detected at 405 nm. The cholesterol esterase inhibitory rates of polyphenol samples with gradient concentrations were calculated based on the recorded absorbance values. Orlistat was selected as the positive control, and its absorbance was measured under the same experimental conditions.

Inhibition rate = [1 − (A − B)/(C − D)] × 100%

(A) Sample solution mixed with pancreatic lipase solution. (B) Sample solution mixed with PBS buffer. (C) PBS buffer mixed with pancreatic lipase solution. (D) Pure PBS buffer.

#### 2.4.3. Establishment and Identification of Hyperlipidemic HepG2 Cell Model

The hyperlipidemic cell model was established with minor modifications according to the reference literature [24]. HepG2 cells were cultured in DMEM medium supplemented with 10% fetal bovine serum (FBS) and 1% penicillin–streptomycin solution. Cells were incubated at 37 °C in a humidified incubator with 5% CO_2_, and subcultured every 2–3 days. HepG2 cells at the logarithmic growth phase were collected and seeded into 6-well plates at a density of 2 × 10^5^ cells/well. After incubation for 48 h, cells were divided into the control group and model groups. Cells in each model group were treated with high-glucose DMEM containing 1% penicillin–streptomycin and palmitic acid (PA) at final concentrations of 100, 200, 300, 400 and 500 μmol/L for 24 h. For the control group, cells were cultured in high-glucose DMEM supplemented with 1% penicillin–streptomycin and an equal concentration of PA vehicle solvent. After the successful construction of the hyperlipidemia HepG2 cell model, cells were categorized into 4 groups: negative control group, model group, positive control group and experimental group. The experimental groups were treated with polyphenol extracts at different mass concentrations (1, 3 and 5 mg/mL) for intervention.

#### 2.4.4. Cell Viability Assay

Cell viability was determined by the MTT assay with minor modifications based on a previously reported method [25]. HepG2 cells in the logarithmic growth phase were evenly dispersed at 1 × 10^4^ cells/well in 96-well plates and incubated for 24 h. After cell attachment, cells were treated with different concentrations of polyphenol samples for 24 h. Subsequently, 50 μL of MTT stock solution (1 × MTT) was added into each well and incubated for 4 h at 37 °C. The supernatant was carefully aspirated, and 150 μL DMSO was added to dissolve formazan crystals. The absorbance was measured at 570 nm using a microplate reader. Cell viability was calculated relative to the control group. The experimental results of cell viability are shown in Supplementary Figure S1.

Cell viability (%) = (OD_experimental group_/OD_blank group_) × 100

#### 2.4.5. Oil Red O Staining of HepG2 Cells

The Oil Red O staining was performed according to a previously published method with minor modifications [26]. After drug intervention, the culture medium was discarded, and cell monolayers were rinsed three times with PBS. Subsequently, cells were fixed with 4% paraformaldehyde solution for 30 min. The fixative was removed, and the wells were washed twice with PBS, followed by incubation with 60% isopropanol for 30 s. Subsequently, the isopropanol solution was removed, and Oil Red O washing solution was added and maintained for 20 s. Upon removal of the washing solution, freshly prepared Oil Red O staining solution was supplemented, and cells were stained for 20 min. The staining solution was aspirated and cells were rinsed with Oil Red O washing solution for 30 s. After removal of the washing solution, the wells were repeatedly washed with PBS until excess dye was completely eliminated.

#### 2.4.6. Determination of Intracellular Lipid Content

The determination of intracellular lipid content was performed according to the reported literature with minor modifications [27]. HepG2 cells in the logarithmic growth phase were seeded into 24-well plates and cultured for 24 h. The medium was aspirated, and cells were washed twice with PBS. Subsequently, cells were treated with 300 μmol·L^−1^ PA for 24 h to establish the hyperlipidemia model, while cells in the control group were incubated with complete DMEM medium only. After 24 h of incubation, the PA-containing medium was aspirated, and cells were treated with samples at concentrations of 1, 3 and 5 mg/mL for another 24 h. Cells were stained with Oil Red O staining solution. Intracellular stained lipid droplets were eluted with 60% isopropanol, and the absorbance was measured at 506 nm using a microplate reader.

#### 2.4.7. Determination of Intracellular Lipase Activity

The determination of intracellular lipase activity was performed according to the reported literature with minor modifications [28]. Cells were cultured in 24-well plates and treated with corresponding concentrations of polyphenols and positive control drugs for 24 h. After the intervention, HepG2 cells were rinsed several times with PBS. The cell pellet was collected by centrifugation at 1000 r·min^−1^ for 10 min. After collection, the pellet was resuspended in 0.1 mL PBS and homogenized to achieve cell lysis. The sample was then centrifuged at 12,000 rpm, 4 °C for 10 min, and the supernatant was retained on ice. Lipase activity was determined using a lipase activity assay kit at a detection wavelength of 405 nm. The detection range of this kit is 6–220 nmol/min/mL. Lipase activity was calculated based on the total protein concentration and expressed as U/mg prot.

#### 2.4.8. Determination of Intracellular TC, TG, HDL-C and LDL-C Levels

Cells were cultured in 24-well plates and treated with corresponding concentrations of polyphenols and positive control drugs for 24 h. After the intervention, cells were rinsed several times with PBS and harvested by centrifugation at 1000 r·min^−1^ for 10 min. The cell sediment was collected and resuspended in 0.3 mL PBS, and homogenized for cell lysis. The levels of TC, TG, HDL-C and LDL-C were measured following the manufacturer’s instructions of corresponding assay kits.

#### 2.4.9. Determination of Intracellular Oxidative Stress Levels

Determination of intracellular oxidative stress levels was performed with minor modifications based on a previously reported method [29]. Cells were seeded in 24-well plates, followed by treatment with polyphenols at designated concentrations as well as positive control agents for 24 h. Upon completion of the intervention, cells were rinsed several times with PBS. The intracellular MDA content and the activities of SOD, GSH-PX, and CAT were measured following the manufacturer’s instructions of the corresponding assay kits.

#### 2.4.10. RT-qPCR Analysis of Lipid Metabolism-Related Gene Expression

Total RNA extraction and reverse transcription were performed according to the manufacturer’s instructions of the corresponding kits. The synthesized cDNA was used as the template for qPCR amplification following the protocol of the quantitative real-time PCR kit. The cycling parameters were set as follows: initial denaturation at 95 °C for 1 min, followed by 40 amplification cycles, each consisting of 95 °C for 5 s and 60 °C for 30 s. *TBP* was selected as the reference gene to quantify the mRNA expression levels of lipid metabolism-related genes, including *HMGCR*, *ACC*, *FASN*, *SREBP-1c* and *CYP7A1* (n = 3). The relative expression of target genes was calculated via the 2^−ΔΔCt^ method. Primer sequences are listed in Table 2.

### 2.5. LC-MS/MS Analysis of Polyphenols from SLKDT

The extraction procedure was slightly modified based on a previously published method reported by Alkhudaydi et al. [30]. Briefly, each sample was ground with liquid nitrogen, and 20 mg of the ground sample was transferred into a centrifuge tube containing 400 μL pre-cooled 80% methanol along with two steel beads. The mixture was homogenized via a low-temperature tissue homogenizer. Subsequently, 600 μL of pre-cooled 80% methanol was added, and the suspension was vortexed for 10 s for thorough mixing. The mixture was treated by ice-bath ultrasonication for 20 min, followed by incubation at −20 °C for 1 h. Afterwards, the mixture was centrifuged at 16,000× *g* and 4 °C for 10 min. The obtained supernatant was filtered through a 0.22 μm microporous membrane, and evaporated to dryness with a vacuum concentrator. The dried residue was redissolved in 200 μL 50% methanol solution, and recentrifuged at 20,000× *g* and 4 °C for 15 min. The final supernatant was collected for subsequent UHPLC-MS/MS analysis.

UHPLC conditions: All samples were stored at 4 °C in an autosampler during the whole detection process. Chromatographic separation was performed on a Waters HSS T3 column using a Shimadzu Nexera X2 LC-30AD UHPLC system (Shimadzu Corporation, Kyoto, Japan), with an injection volume of 8 μL, column temperature of 40 °C and flow rate of 200 μL/min. Mobile phase A consisted of ultrapure water containing 0.1% formic acid, and mobile phase B was acetonitrile. The linear gradient elution procedure was optimized as follows: 0–2.5 min, 0% B. 2.5–9 min, 0–30% B. 9–10 min, 30–100% B. 10–15.4 min, maintained at 100% B. 15.4–15.5 min, and 100–0% B. 15.5–18 min, maintained at 0% B.

Mass spectrometry conditions: Metabolite detection was performed on a 5500 QTRAP mass spectrometer (AB SCIEX) under both positive and negative electrospray ionization (ESI) modes. The ESI source parameters were set as follows. Positive ion mode: Ion source temperature, 550 °C; Ion Source Gas 1 (GAS1), 40 psi; Ion Source Gas 2 (GAS2), 50 psi; Curtain Gas (CUR), 35 psi; and Ion Spray Voltage Floating (ISVF), 5500 V. Negative ion mode: Ion source temperature, 550 °C; GAS1, 40 psi; GAS2, 50 psi; CUR, 35 psi; and ISVF, −4500 V. Characteristic target ion pairs were acquired via multiple reaction monitoring (MRM) mode.

### 2.6. Network Pharmacological Analysis

The PubChem database, https://pubchem.ncbi.nlm.nih.gov/ (accessed on 25 April 2026), was used for the preliminary retrieval and identification of candidate compounds. After downloading the two-dimensional (2D) structural files of these compounds, the structures were submitted to the PharmMapper Server, https://lilab-ecust.cn/pharmmapper/index.html (accessed on 25 April 2026), to predict their potential biological targets. To ensure the reliability of candidate targets, those with a predicted probability below 0.5 were excluded. The genes corresponding to the retained targets were further retrieved from the UniProt database, https://www.uniprot.org/ (accessed on 25 April 2026). Finally, the metabolite–target interaction network was constructed using Cytoscape 3.10.4 software.

Target genes were uploaded to the STRING database, https://cn.string-db.org/ (accessed on 8 May 2026) for PPI network analysis with *Homo sapiens* selected as the organism and a confidence threshold of 0.900. Isolated nodes were eliminated to generate the final PPI network. The network data were then imported into Cytoscape for topological analysis. Cluster analysis of the PPI network was carried out via the CytoNCA plugin to identify core targets. Cytoscape was used for data visualization, and degree values were adopted to regulate node styles for better graphical presentation.

The core targets obtained were imported into Metascape, https://metascape.org/gp/index.html (accessed on 8 May 2026), with the species set to “*Homo sapiens*” for Gene Ontology (GO) functional enrichment analysis and Kyoto Encyclopedia of Genes and Genomes (KEGG) pathway enrichment analysis. The top-ten enriched GO terms and top-twenty enriched KEGG pathways were screened from the resulting data, followed by visualization and output using the online bioinformatics platform https://www.bioinformatics.com.cn/ (accessed on 8 May 2026).

### 2.7. Molecular Docking

Molecular docking was performed to evaluate intermolecular binding affinity between metabolites and target proteins, thereby validating network pharmacology findings. Ligand 3D structures of qualified metabolites were obtained from the PubChem database. The top five core PPI targets were chosen as receptors, whose human protein crystal structures were acquired from the PDB database, https://www.rcsb.org/ (accessed on 10 May 2026), under screening criteria: intrinsic small-molecule ligand binding and structural resolution < 0.25 nm. Receptor pretreatment, including dehydration and polar hydrogenation, was conducted using PyMOL 2.5. AutoGrid 4.2.6 was applied to identify valid protein binding pockets for subsequent docking analysis. AutoDock, https://autodock.scripps.edu/ (accessed on 10 May 2026) was used to calculate ligand–receptor binding free energy, and the optimal docking conformation with the minimum binding energy was visualized by PyMOL 2.5. Binding energy < 0 kcal/mol suggests spontaneous ligand–receptor binding, and binding energy < −5 kcal/mol demonstrates strong binding capacity.

### 2.8. Statistical Analysis

Data analysis was performed using SPSS Statistics 26.0 software. One-way analysis of variance (One-way ANOVA) was applied to compare differences among groups. All data were expressed as mean ± standard deviation (mean ± SD). A value of *p* < 0.05 was considered statistically significant. GraphPad Prism 9.5 software was used for plotting all statistical figures.

## 3. Results and Discussion

### 3.1. Optimization of the Polyphenol Extraction Process from SLKDT

**Figure 1:**
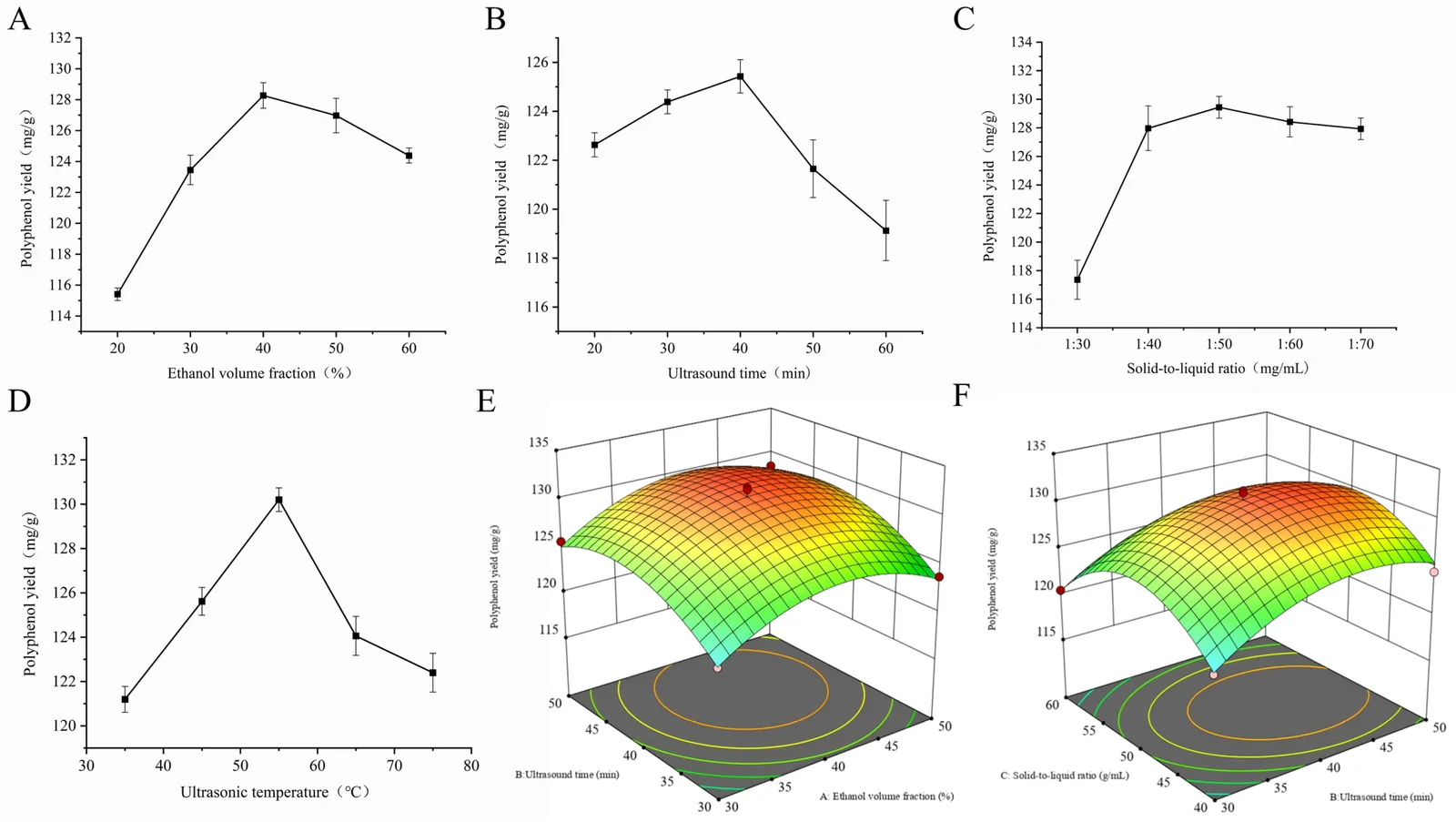

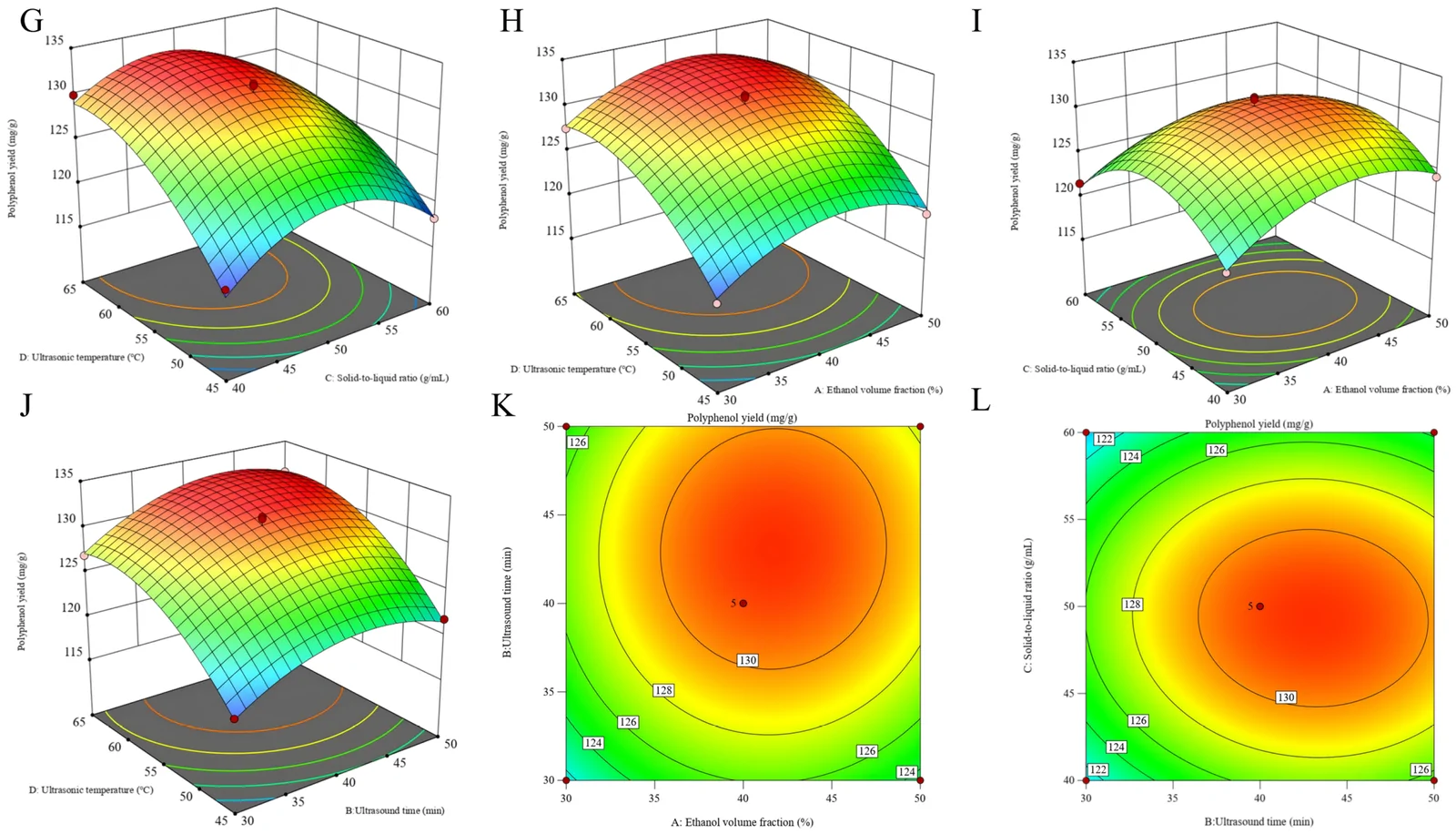

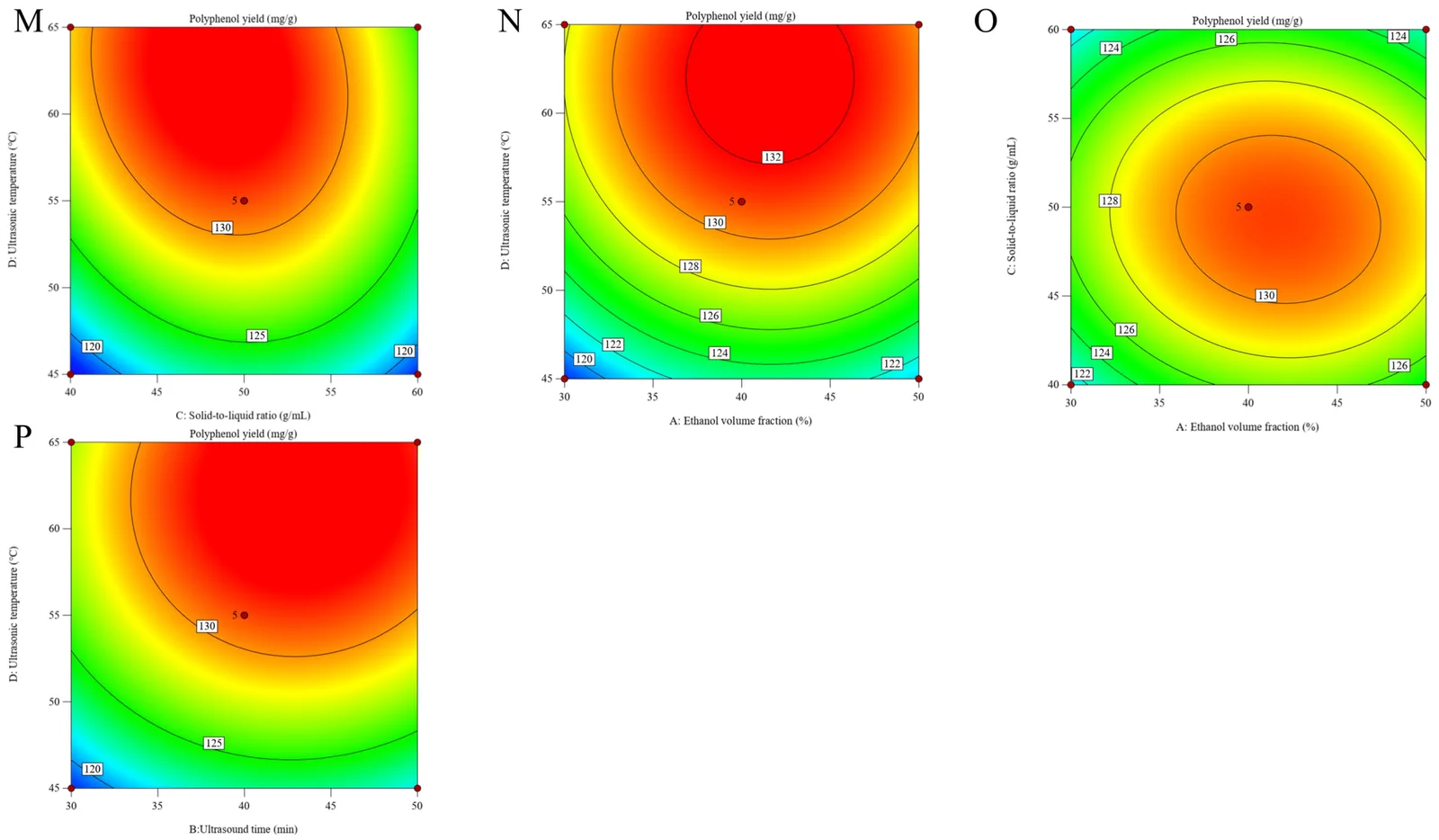
Optimization of extraction process of polyphenols from SLKDT. (**A**) Effect of ethanol volume fraction on the yield of polyphenols, (**B**) effect of ultrasound time on the yield of polyphenols, (**C**) effect of solid-to-liquid ratio on the yield of polyphenols, (**D**) effect of ultrasonic temperature on the yield of polyphenols, (**E**,**K**) effect of ethanol volume fraction and ultrasound time on polyphenol yield, (**F**,**L**) effects of ultrasound time and solid-to-liquid ratio on the yield of polyphenols, (**G**,**M**) effect of solid-to-liquid ratio and ultrasonic temperature on the yield of polyphenols, (**H**,**N**) effect of ethanol volume fraction and ultrasonic temperature on the yield of polyphenols, (**I**,**O**) effect of ethanol volume fraction and solid-to-liquid ratio on the yield of polyphenols, and (**J**,**P**) effect of ultrasound time and ultrasonic temperature on the yield of polyphenols.

Figure 1 shows the outcomes of single-factor tests and response surface optimization. All four variables (ethanol volume fraction, ultrasound time, solid-to-liquid ratio and ultrasonic temperature) showed significant impacts on the polyphenol extraction yield of SLKDT in single-factor trials. For each factor, the yield of polyphenols increased markedly at first and then decreased. The peak yields under individual single-factor optimums were 128.27 mg/g (40% ethanol), 125.42 mg/g (ultrasound time 40 min), 129.25 mg/g (solid-to-liquid ratio 1:50 g/mL), and 129.60 mg/g (ultrasonic temperature 55 °C), respectively. Therefore, the response surface experimental design was formulated on the basis of the single-factor experimental results (Table 3). Response surface data analysis and model fitting were performed using Design-Expert 12 software, yielding a multiple linear regression equation that quantified the influences of the investigated factors on the polyphenol extraction yield of SLKDT. The corresponding regression equation was shown as follows: Y = 131.05 + 1.15A + 1.8875B − 0.629167C + 4.70833D + 0.1875AB − 0.5625AC − 0.025AD − 0.25BC + 0.275BD − 1.175CD − 3.53333A^2^ − 3.11458B^2^ − 5.23958C^2^ − 3.34583D^2^.

As shown in Table 4, the quadratic multiple regression model for the polyphenol yield of SLKDT presented a high F-value of 68.57 with *p* < 0.0001, demonstrating that the established model was statistically extremely significant and possessed good overall reliability. The lack-of-fit *p*-value was 0.7051 (*p* > 0.05), indicating a non-significant lack of fit and a high consistency between the predicted and experimental values. The determination coefficient (R^2^ = 0.9856) and adjusted determination coefficient (R^2^ = 0.9713) reflected that over 97.13% of the data variability could be explained by the established model. Meanwhile, a low coefficient of variation (CV = 0.63%) verified the high precision, stability, and repeatability of the experimental data. Collectively, this model is reliable and suitable for the prediction and optimization of the ultrasonic extraction process of polyphenols from SLKDT. The F-value derived from the ANOVA analysis can directly reflect the magnitude of each factor’s effect on polyphenol yield, where a higher F-value corresponds to a more pronounced influence. Based on the F-value, the influencing order of the four investigated factors on the polyphenol yield of SLKDT was determined as follows: ultrasonic temperature (D) > ultrasound time (B) > ethanol volume fraction (A) > solid-to-liquid ratio (C). Further significance analysis showed that ethanol volume fraction (A), ultrasound time (B), and ultrasonic temperature (D) exerted highly significant effects on polyphenol yield, while the solid-to-liquid ratio (C) exhibited the weakest influence among the four variables.

In recent years, ultrasonic extraction has attracted extensive research attention due to its short extraction time, high efficiency and low cost [31,32]. Both et al. found that ultrasonic extraction increased the content of polyphenols in black tea by about 15% [33]. What is more, ultrasonic extraction not only remarkably elevates phenolic contents, but also favorably retains the functional characteristics of raw materials [34,35]. Therefore, we employed ultrasound-assisted extraction to prepare polyphenols from SLKDT. Based on the established regression model, the optimal ultrasonic extraction conditions for polyphenols from SLKDT were determined as follows: ethanol volume fraction of 42.856%, ultrasound time of 39.313 min, solid-to-liquid ratio of 1:56.108 g/mL, and ultrasonic temperature of 56.656 °C. Under these optimal conditions, the maximum predicted polyphenol yield was 132.558 mg/g. Considering practical experimental operability, the predicted optimal parameters were appropriately adjusted to feasible experimental conditions: an ethanol volume fraction of 43%, ultrasound time of 40 min, solid-to-liquid ratio of 1:56 g/mL, and ultrasonic temperature of 57 °C. Under these modified conditions, the actual polyphenol yield of SLKDT was 131.88 mg/g, with a relative error of only 0.68% compared with the model predicted value. The high consistency between the experimental and predicted yields verified that the established regression model was accurate and reliable.

### 3.2. In Vitro Lipid-Lowering Activity

#### 3.2.1. The Inhibitory Effects of Pancreatic Lipase and Cholesterol Esterase

**Figure 2:**
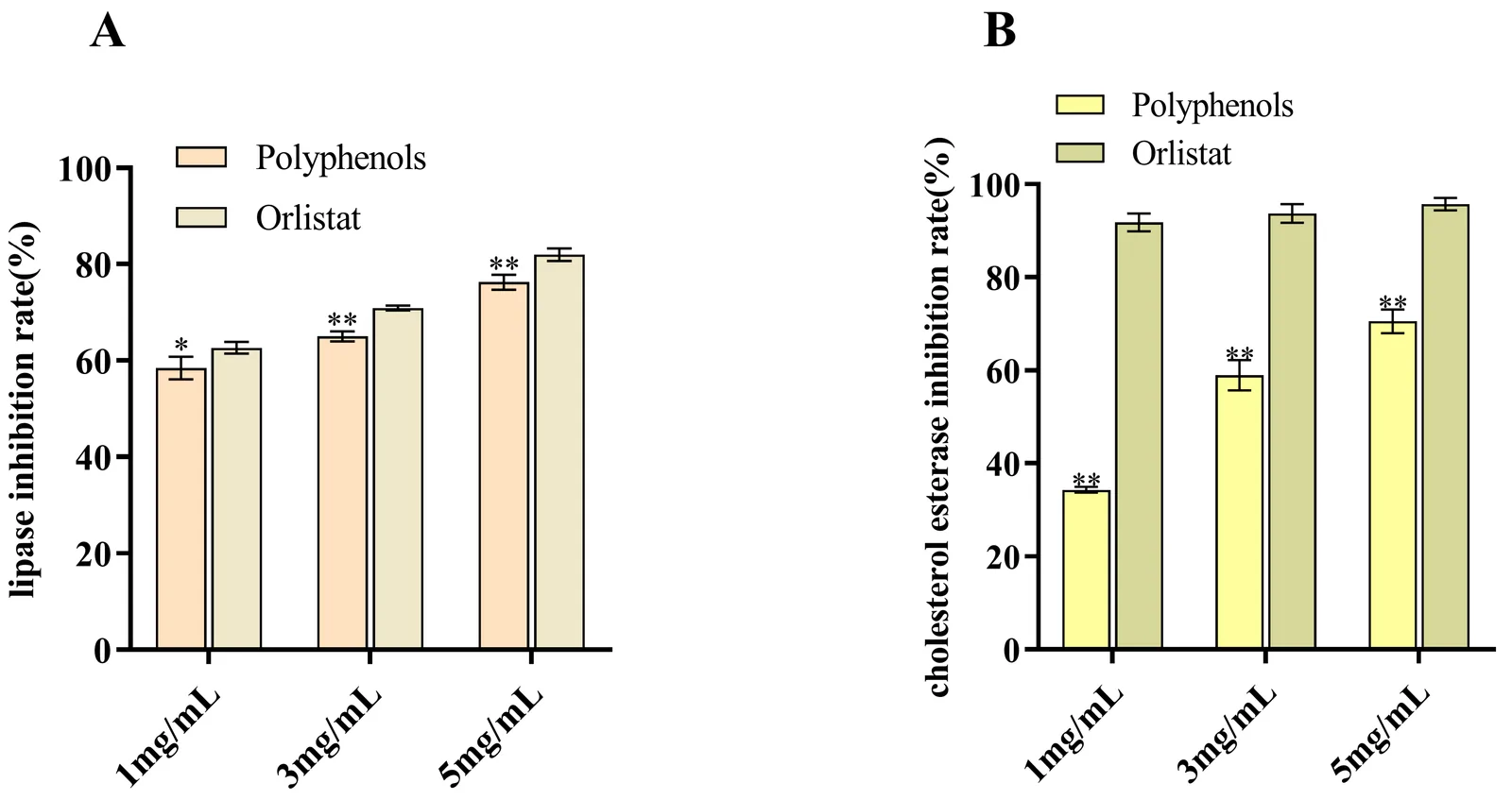
Inhibitory effects of SLKDT polyphenols on pancreatic lipase and cholesterol esterase. (**A**) Pancreatic lipase inhibition rate; (**B**) cholesterol esterase inhibition rate. * indicates *p* < 0.05 compared with positive orlistat; ** indicates *p* < 0.01 compared with positive orlistat.

Pancreatic lipase is a key enzyme in the process of digestion. The inhibition of pancreatic lipase activity can effectively suppress the digestion and absorption of dietary lipids, reduce systemic lipid accumulation, and thereby mitigate the risk of obesity-related metabolic disorders. The inhibitory effects of natural polyphenols on pancreatic lipase have been well documented in previous studies [36,37]. Martinez-Gonzalez et al. [38] demonstrated that multiple phenolic compounds, including caffeic acid, p-coumaric acid, and quercetin, exert varying degrees of inhibitory effects on pancreatic lipase. In addition, tea-derived products and their phenolic constituents have also been validated as effective pancreatic lipase inhibitors [39]. As presented in Figure 2A, polyphenols from SLKDT exhibited inhibitory effects against pancreatic lipase in this study. Especially at medium and high concentrations, a significant inhibitory effect was observed (*p* < 0.01), reaching as high as 76.26%. This finding is consistent with the results reported by Gao et al. [22]. regarding the inhibitory activity of polyphenols against pancreatic lipase. More importantly, our polyphenolic extract possessed stronger inhibitory capacity under equivalent concentration conditions.

Orlistat, the positive control drug, also exhibited concentration-dependent lipase-inhibitory activity, and its inhibitory potency was slightly higher than that of SLKDT polyphenols at all tested concentrations. This observation is consistent with numerous in vitro studies on plant polyphenols against pancreatic lipase: natural polyphenols such as coffee leaf-derived polyphenols and polyphenols extracted from black tea can effectively suppress pancreatic lipase, yet most natural polyphenols show weaker lipase-inhibitory effects than the synthetic lipase inhibitor orlistat [36,40]. Mechanistically, orlistat specifically binds to the hydrophobic active-site pocket of pancreatic lipase and forms stable covalent bonds with the serine residue (Ser153), thereby exerting irreversible inhibition [41]. In contrast, polyphenols mostly bind to enzyme proteins via non-covalent bonds and hydrophobic interactions, disrupting the spatial conformation of enzymes and altering the microenvironment surrounding internal tryptophan residues, which corresponds to reversible inhibition. Furthermore, the inhibitory potency of polyphenols is closely related to the number of phenolic hydroxyl groups, galloyl moieties, degree of polymerization and glycosylation status of molecules [13,42]. This accounts for the generally inferior lipase-inhibitory performance of natural polyphenols compared with orlistat. Meanwhile, significant pancreatic lipase-inhibitory effects (*p* < 0.05) were achieved by the SLKDT polyphenol fraction at a relatively low concentration (1 mg/mL) in the present study, implying that this fraction contains highly active lipase-inhibitory monomers such as chrysin. It has been reported that such compounds can insert into the lid domain of pancreatic lipase and block substrate access to the catalytic pocket [43], which is consistent with the screening results of core bioactive components in subsequent experiments.

Besides pancreatic lipase, cholesterol esterase is another pivotal enzyme participating in the lipid digestion and absorption pathway. The inhibition of cholesterol esterase can effectively suppress cholesterol absorption in vivo. The inhibitory effect of polyphenols from SLKDT on cholesterol esterase is shown in Figure 2B. The inhibitory rate against cholesterol esterase gradually increased with the rising sample concentration. The inhibition rate reached 34.29% at 1 mg/mL and 70.53% at 5 mg/mL, demonstrating an obvious concentration-dependent inhibitory trend, which was consistent with the findings of Ngamukote et al. [44] and Rahim et al. [45], who reported that gallic acid and epicatechin possessed stronger pancreatic lipase-inhibitory capacity among various phenolic compounds. Our LC-MS/MS identification confirmed that both epicatechin and gallic acid existed in the polyphenols of SLKDT. This may partially account for the potent cholesterol esterase inhibitory capacity of SLKDT polyphenols.

#### 3.2.2. Establishment of PA-Induced Hyperlipidemia Model in HepG2 Cells

**Figure 3:**
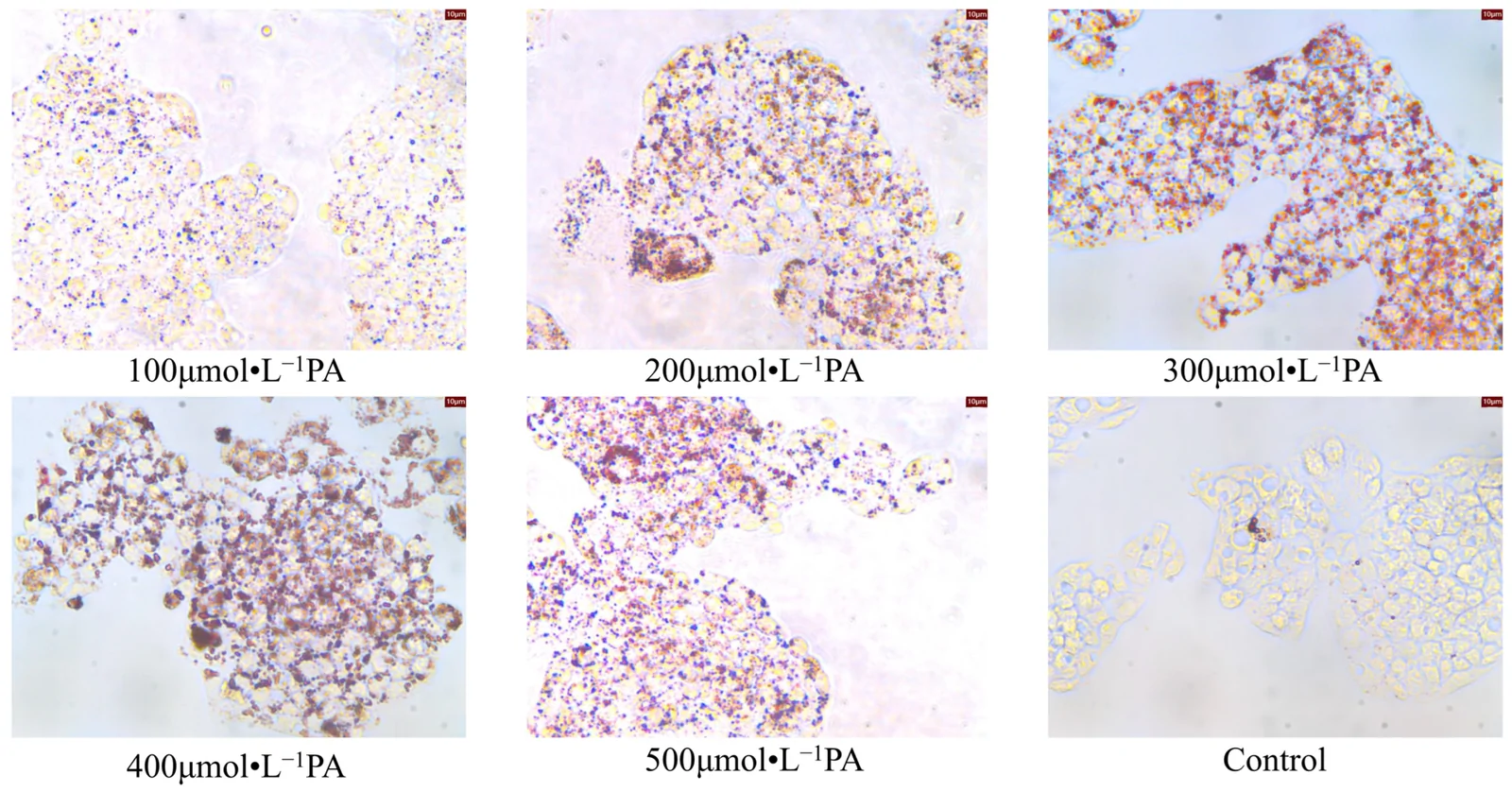
Staining results of HepG2 cells induced by different concentrations of PA (400×).

To further explore the lipid-lowering activity of SLKDT polyphenols, a palmitic acid (PA)-induced hyperlipidemia model was established in HepG2 cells (Figure 3). The PA concentrations used for cell model induction were set at 100, 200, 300, 400, and 500 μmol·L^−1^. ORO staining results demonstrated that HepG2 cells in the normal control group presented clear cell boundaries and typical islet-like morphology, with no obvious red-stained lipid droplets. In contrast, HepG2 cells treated with different PA concentrations exhibited a significant accumulation of intracellular lipid droplets, as evidenced by extensive red staining. These observations are consistent with previous ORO staining findings reported by Yao et al. [8] and Cui et al. [24] Based on the comprehensive staining results, 300 μmol·L^−1^ PA was selected as the optimal concentration for subsequent cell model construction.

#### 3.2.3. Effect of Polyphenols in SLKDT on PA-Induced Lipid Accumulation in HepG2 Cells

**Figure 4:**
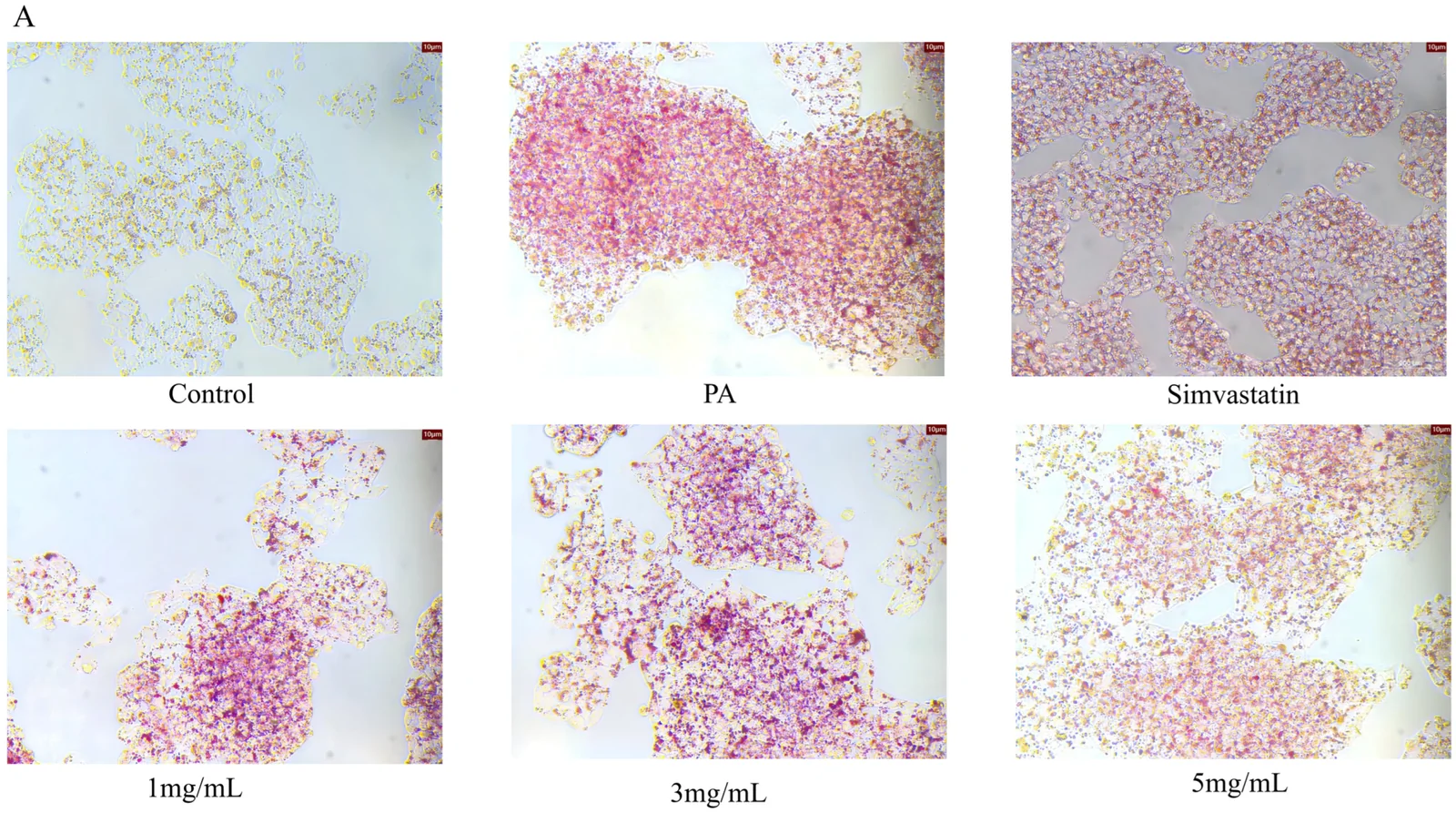

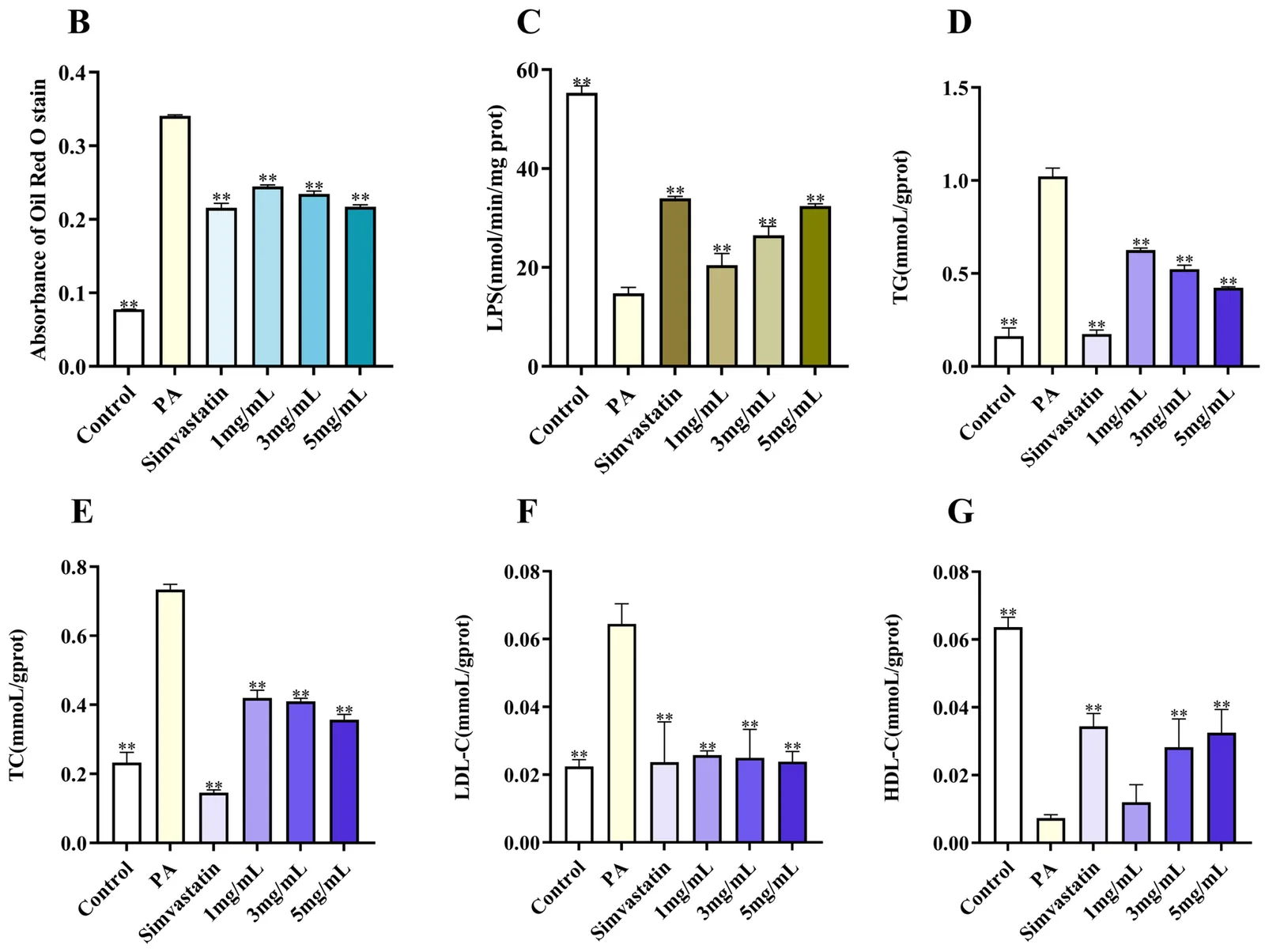

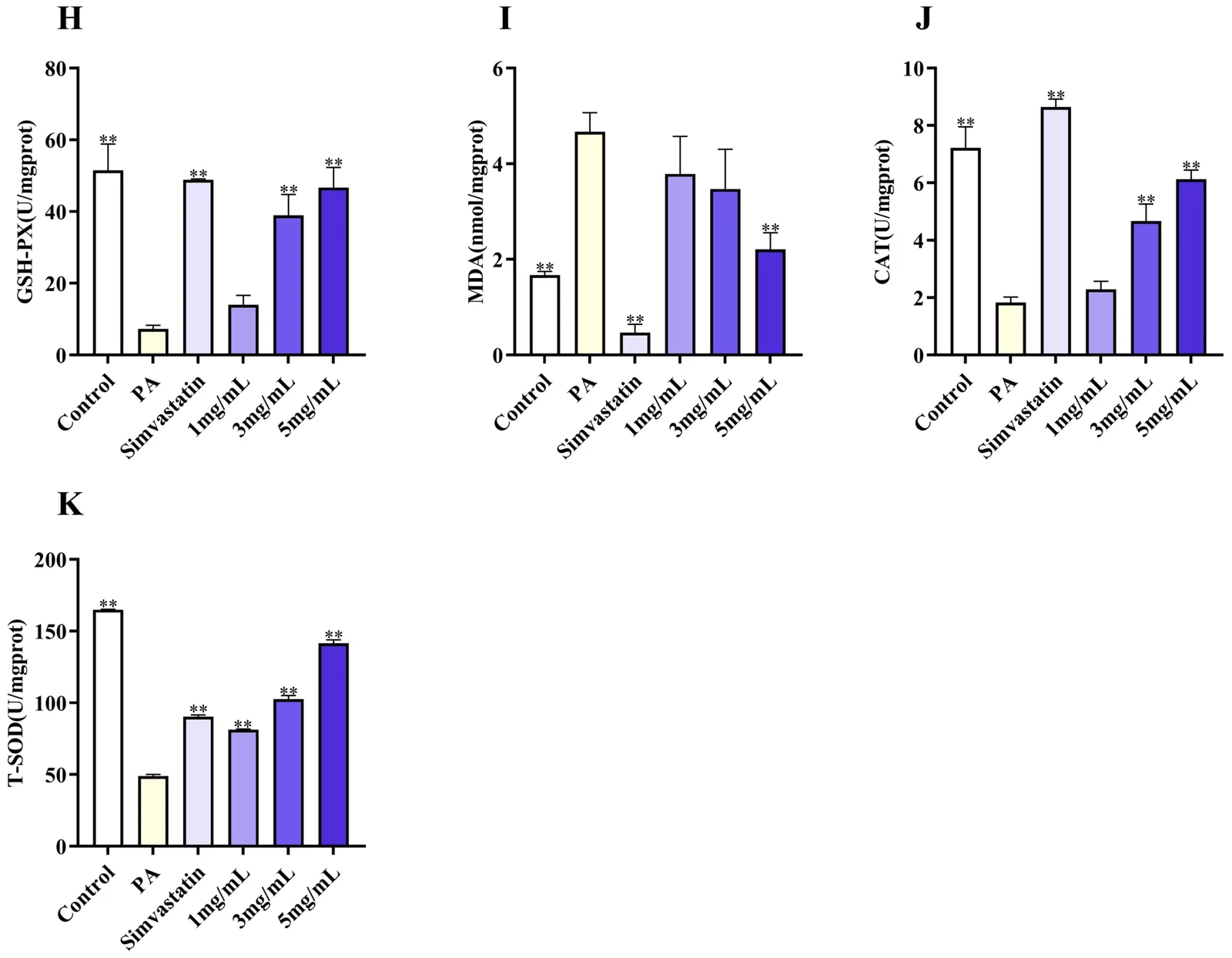
Regulatory effects of polyphenols from SLKDT on lipid accumulation and oxidative stress in HepG2 cells. (**A**) Image of cells stained with polyphenol Oil Red O of SLKDT (200×). (**B**) Intracellular lipid content. (**C**) Intracellular LPS activity. (**D**) Intracellular TG content. (**E**) Intracellular TC content. (**F**) Intracellular LDL-C content. (**G**) Intracellular HDL-C content. (**H**) Intracellular GSH-PX activity. (**I**) Intracellular MDA content. (**J**) Intracellular CAT activity. (**K**) Intracellular T-SOD activity. ** indicates *p* < 0.01 compared with the model group.

Hyperlipidemia is clinically characterized by elevated serum levels of TC, TG and LDL-C, along with a decreased level of HDL-C. To investigate the regulatory effects of SLKDT polyphenols on intracellular lipid accumulation in HepG2 cells, intracellular lipid content, lipase activity, and multiple serum lipid indicators, including TG, TC, HDL-C, and LDL-C, were determined via Oil Red O staining. ORO staining results (Figure 4A) showed that PA induction significantly increased the number of intracellular lipid droplets, with abundant red-stained lipid droplets clearly observed in the model group. Compared with the PA model group, the accumulation of intracellular lipid droplets was markedly reduced in cells treated with low, medium, and high concentrations of SLKDT polyphenols, as well as in the simvastatin-positive group, demonstrating that SLKDT polyphenols can effectively alleviate intracellular lipid deposition.

Quantitative analysis of intracellular lipid content further verified the above phenotypic changes (Figure 4B). PA induction dramatically increased intracellular lipid accumulation, whereas intervention with SLKDT polyphenol extracts reversed this abnormality. Notably, medium and high concentrations of polyphenols significantly reduced intracellular lipid content compared with the PA group (*p* < 0.01), confirming the excellent lipid-lowering efficacy of SLKDT polyphenols. Intracellular lipolysis capacity is highly dependent on lipase activity. As shown in Figure 4C, lipase activity was significantly inhibited after PA treatment, which impaired cellular lipolysis function and ultimately led to excessive lipid accumulation in HepG2 cells. In contrast, intervention with SLKDT polyphenols significantly upregulated intracellular lipase activity at all tested concentrations (*p* < 0.01), enhanced cellular lipolysis capacity, and thereby effectively attenuated intracellular lipid accumulation.

Figure 4D–G illustrated the intracellular levels of TG, TC, LDL-C, and HDL-C following SLKDT polyphenol intervention. PA induction significantly increased the contents of TG, TC, and LDL-C, while markedly decreasing HDL-C levels in HepG2 cells. Treatment with SLKDT polyphenols effectively reversed PA-induced dyslipidemia. Specifically, medium and high concentrations of polyphenols significantly upregulated HDL-C content (*p* < 0.01). Notably, 5 mg/mL SLKDT ameliorated multiple lipid parameters to an extent comparable to or even reaching that of the simvastatin-positive control, indicating robust hypolipidemic activity. Simvastatin, as an HMG-CoA reductase inhibitor, exerts its effects primarily by suppressing cholesterol biosynthesis [46], whereas SLKDT, as a natural polyphenol-rich extract, may mediate its lipid-lowering actions through more pleiotropic mechanisms. In addition to inhibiting cholesterol synthesis, these mechanisms may also involve antioxidant activity, anti-inflammatory effects, and the modulation of lipid metabolism-related signaling pathways, which act synergistically to exert its hypolipidemic effects. The lipid-regulating profile observed in the present study is highly consistent with previous literature reports. Yao et al. [8] verified that highland barley polyphenol extracts markedly reduced intracellular TG, TC and LDL-C levels in hepatocytes. Likewise, Huang et al. [47] reported that polyphenol extracts from *Ilex polycarpa* effectively alleviated oleic acid (OA)-triggered lipid accumulation in cells. Furthermore, an in vivo animal study conducted by Wu et al. [48] using high-fat diet-induced hyperlipidemic mice demonstrated that SLKDT extracts ameliorated dyslipidemia by decreasing serum TC, TG and LDL-C concentrations while elevating HDL-C levels. Accumulating evidence has illustrated that polyphenolic compounds serve as the core bioactive substances responsible for the anti-hyperlipidemic and anti-obesity properties of tea [49]. Chlorogenic acid and its derivatives are characteristic phenolic constituents of SLKDT, which exert crucial functions in obesity prevention and the modulation of lipid metabolism [48]. Based on the LC-MS/MS analysis results of this study, chlorogenic acid is identified as a major phenolic compound with relatively high content in the polyphenols of SLKDT, suggesting that this component may be a key factor responsible for its lipid-lowering activity.

#### 3.2.4. Effects of Polyphenols in SLKDT on Oxidative Stress Induced by PA in HepG2 Cells

Oxidative stress arises from an imbalance between oxidants and antioxidants, leading to excess oxidants that oxidize lipids, proteins, enzymes, and DNA [50]. The PA-triggered oxidative stress in HepG2 cells upon treatment with SLKDT polyphenols is illustrated in Figure 4H–K. Compared with the control group, the model group exhibited markedly reduced activities of GSH-PX, CAT, and T-SOD, as well as significantly elevated MDA content. GSH-PX, SOD, and CAT represent pivotal enzymatic components of the cellular antioxidant defense system. SOD catalyzes the dismutation of superoxide anion radicals into hydrogen peroxide, which is subsequently decomposed into water and oxygen by CAT and GSH-PX. Collectively, these three enzymes synergistically scavenge ROS and maintain intracellular redox homeostasis [51]. Following PA treatment, the activities of these enzymes were significantly decreased, resulting in impaired ROS clearance, exacerbated lipid peroxidation, increased MDA production, and ultimately aggravated cellular damage. Intervention with SLKDT polyphenols significantly ameliorated all oxidative stress parameters in a dose-dependent manner, as evidenced by the restored activities of GSH-PX, CAT, and T-SOD and the reduced MDA content. These findings were consistent with a previous study by Liu et al. [19] which reported the regulatory effect of SLKDT polyphenols on D-galactose-induced oxidative stress in mice. In summary, the present study demonstrates that SLKDT effectively ameliorates PA-induced oxidative stress injury by restoring antioxidant enzyme activities and reducing lipid peroxidation product generation. These effects, in synergy with its lipid-lowering activity, collectively underlie its protective role against cellular lipid metabolism disorders.

#### 3.2.5. Gene Expression in HepG2 Cells Induced by PA

**Figure 5:**
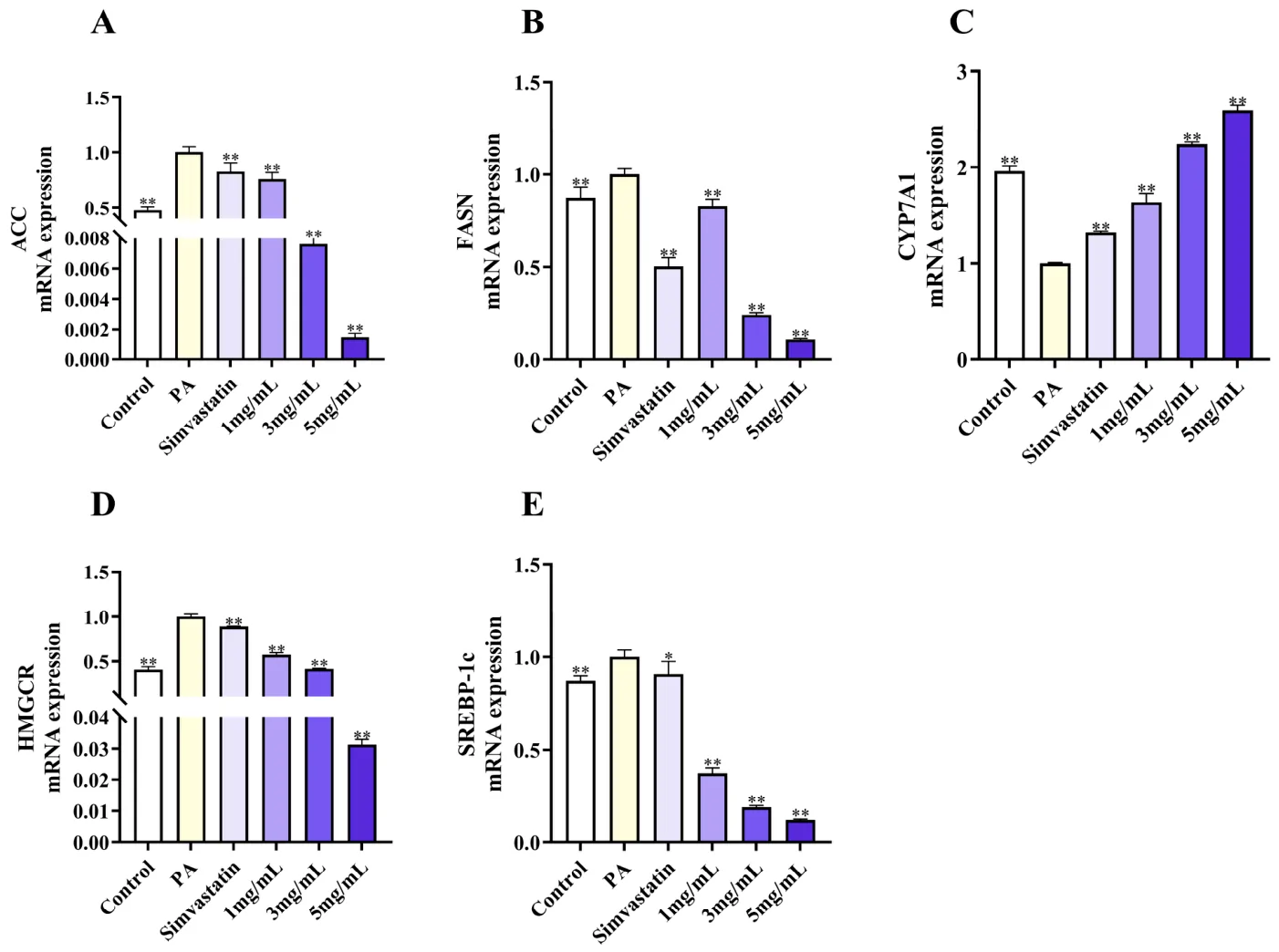
Regulatory effects of SLKDT polyphenols on key genes involved in lipid metabolism in HepG2 cells. (**A**) *ACC* mRNA expression. (**B**) *FASN* mRNA expression. (**C**) *CYP7A1* mRNA expression. (**D**) *HMGCR* mRNA expression. (**E**) *SREBP-1c* mRNA expression. * indicates *p* < 0.05 compared with the model group; ** indicates *p* < 0.01 compared with the model group.

To further explore the potential lipid-lowering mechanism of SLKDT polyphenols, RT-qPCR was performed to detect the mRNA expression of key lipid metabolism-related genes, including *ACC*, *FASN*, *HMGCR*, *SREBP-1c*, and *CYP7A1*. As shown in Figure 5, the mRNA levels of *ACC*, *FASN*, *HMGCR*, and *SREBP-1c* were significantly upregulated in the PA-stimulated model group compared with the normal control group. Similar to the positive drug simvastatin, SLKDT polyphenols markedly downregulated the expression of *ACC*, *HMGCR* and *SREBP-1c* in a clear dose-dependent manner. In contrast, PA intervention significantly suppressed the transcriptional level of *CYP7A1*, whereas SLKDT polyphenol treatment effectively reversed this trend and dramatically upregulated *CYP7A1* expression (*p* < 0.01). These results are consistent with previous studies reported by Wan et al. [52] and Ilias et al. [53].

*ACC* and *FASN* are critical rate-limiting enzymes involved in endogenous fatty acid biosynthesis, and their overexpression accelerates excessive fatty acid production, ultimately resulting in intracellular lipid accumulation. As the core upstream transcription factor governing lipogenesis, *SREBP-1c* directly targets and activates the transcription of *ACC* and *FASN* [54]. Accordingly, the elevated *SREBP-1c* expression in the PA model contributes substantially to the upregulation of downstream *ACC* and *FASN*, which aggravates lipid synthesis and deposition in HepG2 cells. In this study, SLKDT polyphenols concentration-dependently inhibited the mRNA expression of *SREBP-1c*, *ACC*, and *FASN*, indicating that SLKDT polyphenols ameliorate PA-induced aberrant lipogenesis via suppressing the *SREBP-1c*-mediated fatty acid synthesis pathway. This regulatory pattern is in line with a previous study, which demonstrated that the hypolipidemic effect of apigenin relies on the downregulation of *SREBP-1c* signaling [55]. *HMGCR* acts as the rate-limiting enzyme of cholesterol synthesis, whose expression level directly determines the rate of intracellular cholesterol anabolism. In comparison, *CYP7A1* is a key catalytic enzyme responsible for converting cholesterol to bile acids, and its insufficient expression blocks cholesterol catabolism and excretion. The simultaneous upregulation of *HMGCR* and downregulation of *CYP7A1* in PA-treated HepG2 cells confirmed the occurrence of cholesterol metabolic disorder, characterized by enhanced cholesterol synthesis and impaired cholesterol clearance. After intervention with SLKDT polyphenol, *HMGCR* transcription was significantly inhibited while *CYP7A1* mRNA expression was prominently restored. This finding suggested that SLKDT polyphenols improved PA-disrupted cholesterol homeostasis through dual regulation: inhibiting cholesterol biosynthesis and facilitatating cholesterol–bile acid conversion. Studies have demonstrated that black tea can alleviate hyperlipidemia by modulating bile acid circulation, which is consistent with our findings [56]. In addition, the above results were supported by the research of Long et al. [57] and Ilias et al. [53]. Collectively, the in vitro experiments clearly demonstrated that SLKDT exerts significant hypolipidemic activity and ameliorates lipid metabolism disorders. However, the active constituents and underlying molecular regulatory networks remain largely unknown. Therefore, building upon the validated lipid-lowering effects, the present study performed LC-MS/MS profiling and network pharmacology analysis to systematically identify the bioactive compounds, core targets, and key signaling pathways mediating the hypolipidemic effects of SLKDT.

### 3.3. Analysis of Polyphenols in SLKDT by LC-MS/MS

In this study, polyphenolic components of SLKDT were analyzed using LC-MS/MS. Eighty-eight high-abundance compounds were preliminarily screened according to the magnitude of their relative peak areas, which were categorized into five groups: glycosides, flavonoids, phenols, phenolic acids, and other components (Table 5). Specifically, 30 glycosides, 30 flavonoids, 12 phenolic compounds, 13 phenolic acids, and three other compounds were detected. Glycosides were the most abundant components, followed by flavonoids. The identified phenolic compounds were consistent with those previously reported in *Argania spinosa* fruits by Khallouki et al. [58], most of which possess potent anti-inflammatory and antioxidant activities [59,60]. In addition, major constituents, including caffeic acid, epicatechin, and isohyperoside, were detected in the present SLKDT polyphenol extract, which was consistent with the findings of polyphenol extracts from barley reported by Yao et al. [8].

Glycosides constituted the most abundant polyphenol subclass in SLKDT, with distinctly higher peak areas than other compounds. Notably, the top four most abundant compounds identified in this study were all glycoside derivatives. Galangin-7-O-glucoside exhibited the highest peak area among all identified constituents, reaching 218,413,260.10 ± 6,288,083.23, which indicated that it was the predominant core glycosylated active compound in SLKDT polyphenols. Apigenin-7-O-neohesperidoside showed a peak area of 184,912,260.77 ± 583,901.07, with a relative content inferior only to galangin-7-O-glucoside. Kaempferol-3-O-glucoside had a peak area of 180,369,410.50 ± 8,198,167.31, with a comparable abundance to apigenin-7-O-neohesperidoside. Luteolin-7-O-glucoside (161,302,181.60 ± 6,408,722.46) was also identified as a high-abundance glycosylated component in the sample. Furthermore, kaempferol-3-O-rutinoside and luteolin-7-O-rutinoside displayed peak areas above 1 × 10^8^, further demonstrating that the polyphenols of SLKDT are predominantly present as glycoside. The highly accumulated flavonoid glycosides identified in this study are structurally derived from three typical aglycone cores: luteolin, quercetin, and kaempferol. These bioactive constituents have been validated to exert prominent antioxidant capacities, along with multiple pharmacological properties, including anti-inflammatory, antibacterial, anti-tumor, cardioprotective, and anti-diabetic activities [61,62,63]. Specifically, kaempferol-3-O-(6″-galloyl) galactoside, orientin-2″-O-galactoside, and acacetin-7-O-glucoside are responsible for hypoglycemic regulation [64,65], whereas acteoside and quercetin-3-O-glucoside exhibit potent lipid metabolism-modulating effects [66,67].

Flavonoids are also dominant constituents of SLKDT polyphenols. In this work, rutin, isohyperoside, and bavachinin were identified as the predominant flavonoid components, with peak areas exceeding 7 × 10^7^. Among them, rutin exhibited the highest abundance. Accumulating evidence has demonstrated that rutin possesses multiple pharmacological properties, including antioxidant, anti-inflammatory, hepatoprotective, gastroprotective, and hypoglycemic activities [68,69]. Liu et al. [70] reported that rutin could alleviate oleic acid-induced lipid accumulation in Hela cells and ameliorate lipid deposition in in vitro and in vivo models of diabetes combined with non-alcoholic fatty liver disease. Isohyperoside has been proven to exert myocardial protective effects by protecting cell membranes from oxidative damage [71]. Bavachinin exhibits anti-angiogenic and anti-tumor activities and can alleviate cerebral ischemia–reperfusion injury in rats via its anti-inflammatory and antioxidant properties [72,73]. In addition to the above components, apigenin, 2′-Hydroxygenistein, and isoscutellarein were also relatively high in content. As a major flavonoid constituent, apigenin displays antioxidant and anti-inflammatory capacities, as well as hypoglycemic, lipid-regulating, anti-apoptotic, anti-cancer, and neuroprotective effects [74,75,76]. 2′-Hydroxygenistein displays stronger antioxidant capacity than other genistein derivatives and effectively inhibits α-glucosidase, indicating promising hypoglycemic potential; it also presents latent anti-osteoporosis activity [77,78,79,80]. Moreover, nicotiflorin identified in this study has been reported to possess hypoglycemic and anti-lipogenic functions [81,82]. It can activate brown adipose tissue via the β3-adrenergic receptor pathway, thereby ameliorating obesity [83]. Isorhamnetin and its glycoside derivatives have also been validated as effective anti-obesity ingredients capable of regulating blood glucose homeostasis [84]. In addition, tiliroside markedly suppresses lipid accumulation, suggesting its crucial role in lipid metabolism modulation [85]. Furthermore, Adachi et al. [86] demonstrated that tiliroside could reduce uric acid production in the liver and modulate renal uric acid reabsorption, thereby exerting a hypouricemic effect.

Phenolics and phenolic acid compounds are widely distributed natural bioactive substances in plants. Chemical analysis of this study revealed that hydroxytyrosol was the dominant compound with the highest peak area among these components, other compounds such as 4-hydroxybenzoic acid, 3,4-dihydroxybenzoic acid, caffeic acid, 2,5-dihydroxybenzoic acid, gentisic acid, 1-O-salicyloyl-D-glucose, 6-O-caffeoyl-D-glucose, syringin, and p-coumaric acid also showed relatively high contents. Additionally, typical active phenolic acids including chlorogenic acid, rosmarinic acid, and gallic acid were detected. These substances serve as the core molecular basis for the antioxidant and anti-inflammatory biological functions of SLKDT polyphenols [87,88,89]. Among them, chlorogenic acid has been proven to exhibit significant hypoglycemic and hypolipidemic activities, playing an important role in regulating glucose and lipid metabolism [90]. Pharmacological studies have confirmed that the high-abundance phenolic and phenolic acid compounds identified in this study exert diverse bioactivities. Specifically, hydroxytyrosol possesses broad-spectrum pharmacological properties, including anti-tumor, antiviral, antibacterial, neuroprotective, cardiovascular protective, and hypoglycemic activities [91,92]. 4-Hydroxybenzoic acid has shown hypoglycemic, antibacterial and neuroprotective effects [93]. Other studies had reported that 3, 4-dihydroxybenzoic acid (Protocatechuic acid) also had anti-diabetes, antibacterial, anti-cancer, and antiviral capacities [94]. Caffeic acid exhibits a wider range of biological functions. Beyond its well-documented antioxidant, anti-inflammatory, immunomodulatory, antibacterial, and neuroprotective effects, caffeic acid participates in glucose metabolism regulation. Accumulated animal experiments have verified its ability to significantly reduce blood glucose levels in mice, suggesting its promising hypoglycemic potential [95,96]. This study also identified three other phenolic compounds, including vanillic acid, taxifolin, and psyllium saponin. Based on the diverse phenolic, phenolic acid, and flavonoid constituents detected in this study and their well-documented pharmacological activities, the multiple bioactive compounds in SLKDT polyphenols possess potential antioxidant, hypoglycemic, and hypolipidemic capacities. Therefore, it is speculated that the superior antioxidant and glucose–lipid regulatory activities of SLKDT polyphenols are closely attributed to its abundant and diverse bioactive components. To further validate this hypothesis, a series of in vitro experiments were subsequently performed to systematically evaluate the biological activities of SLKDT polyphenols.

### 3.4. Compound Network Pharmacological Analysis and Molecular Docking

#### 3.4.1. Screening of Hypolipidemic Targets of Polyphenols from SLKDT

**Figure 6:**
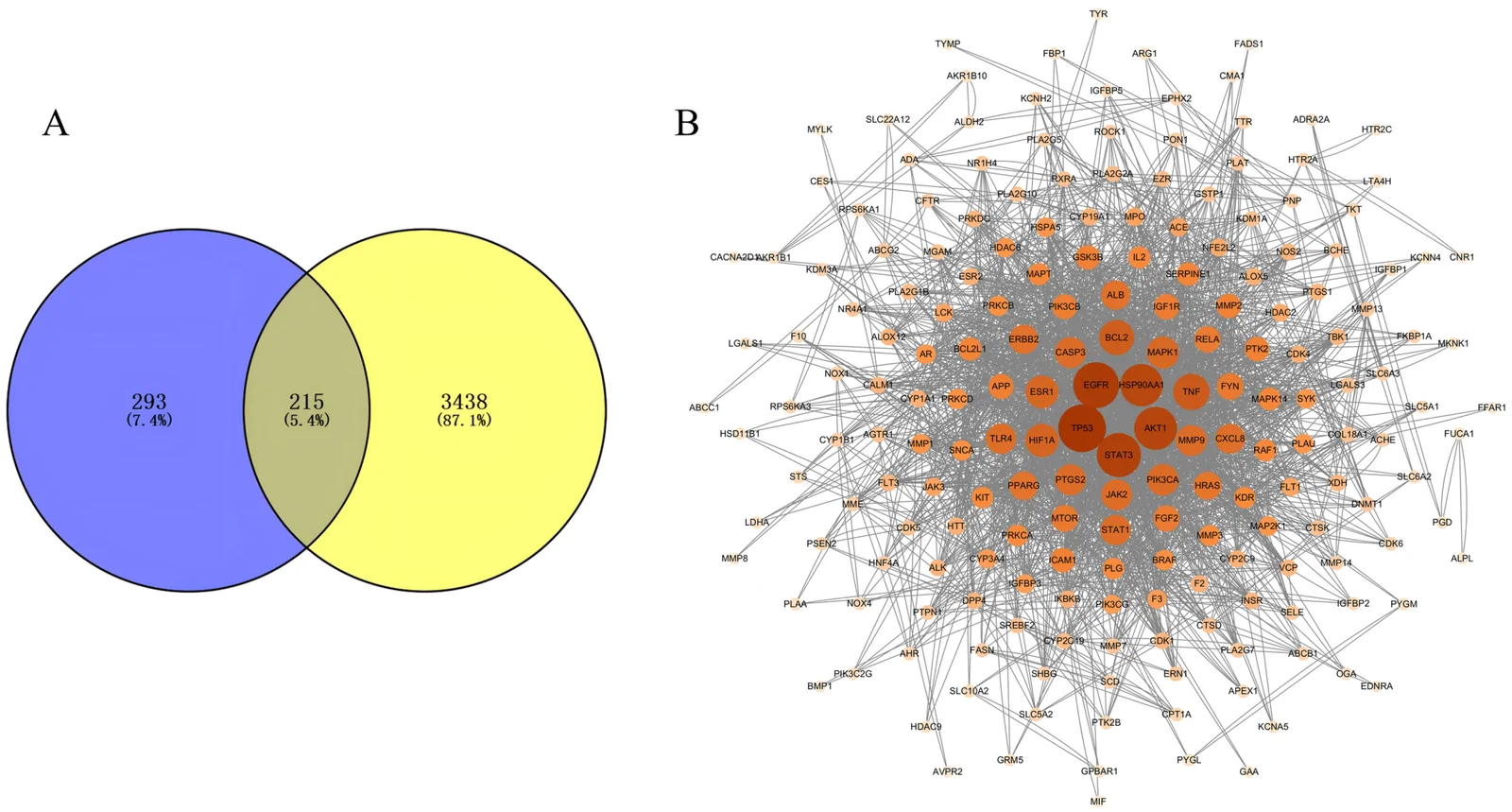

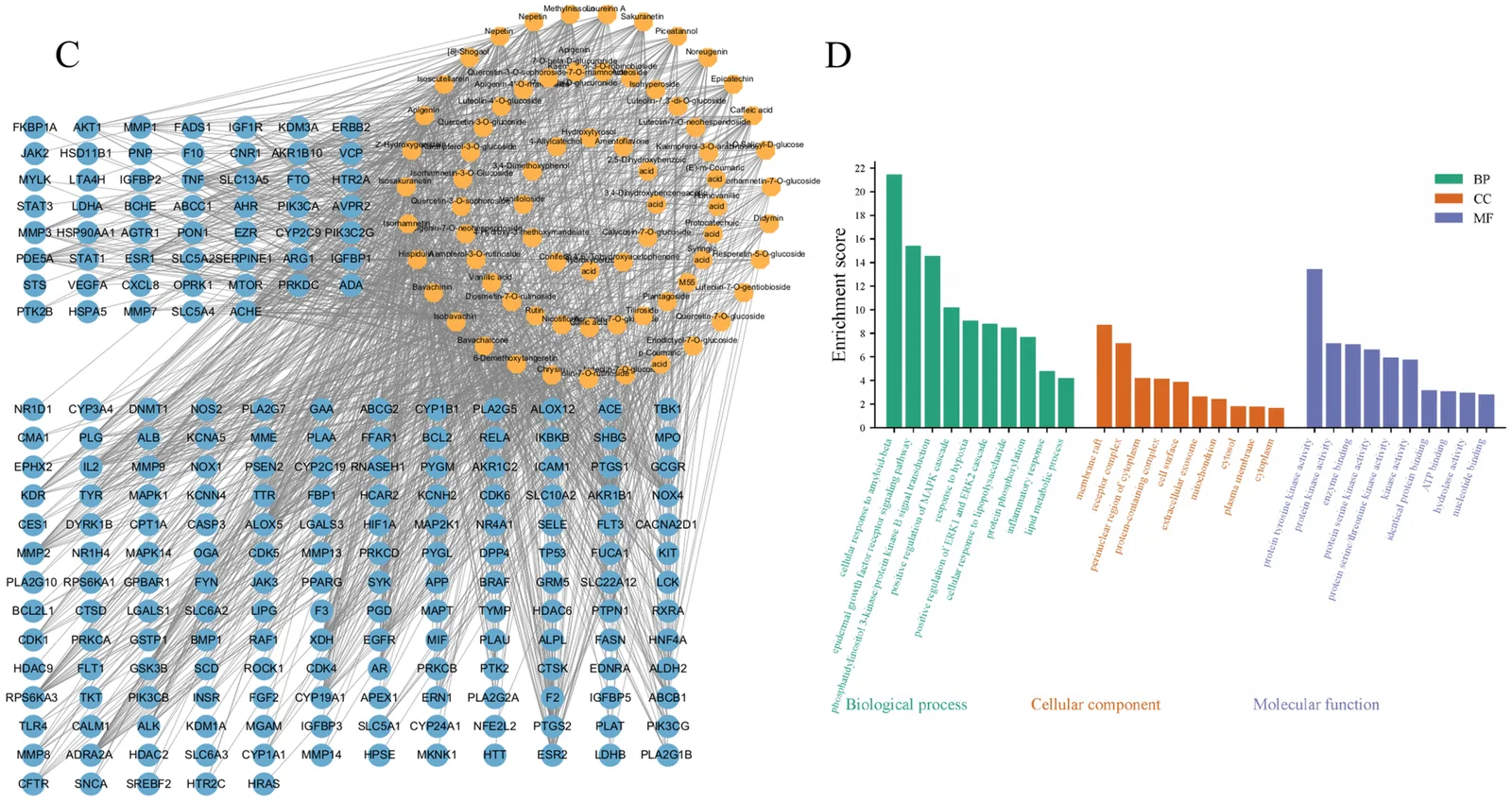

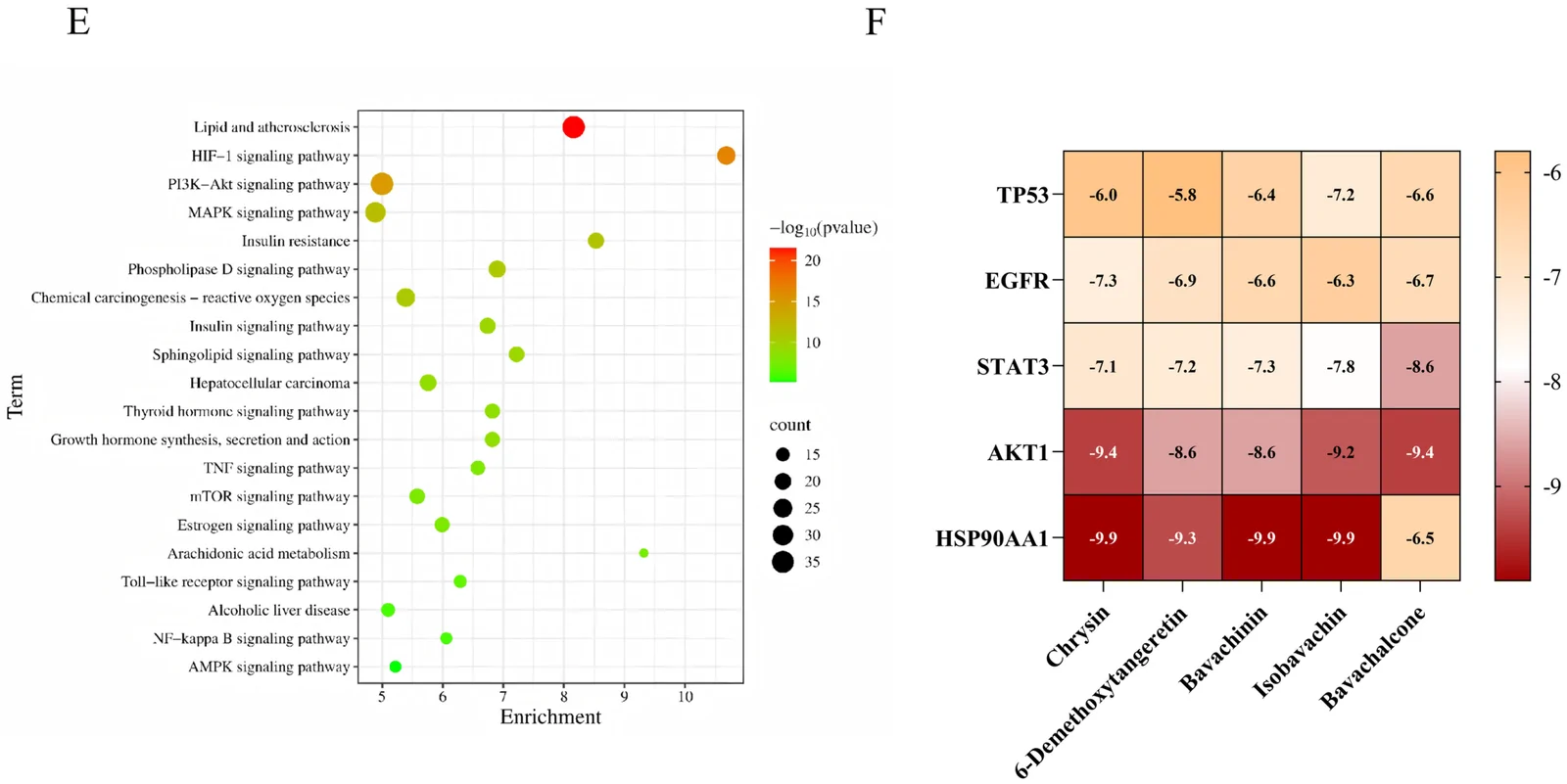

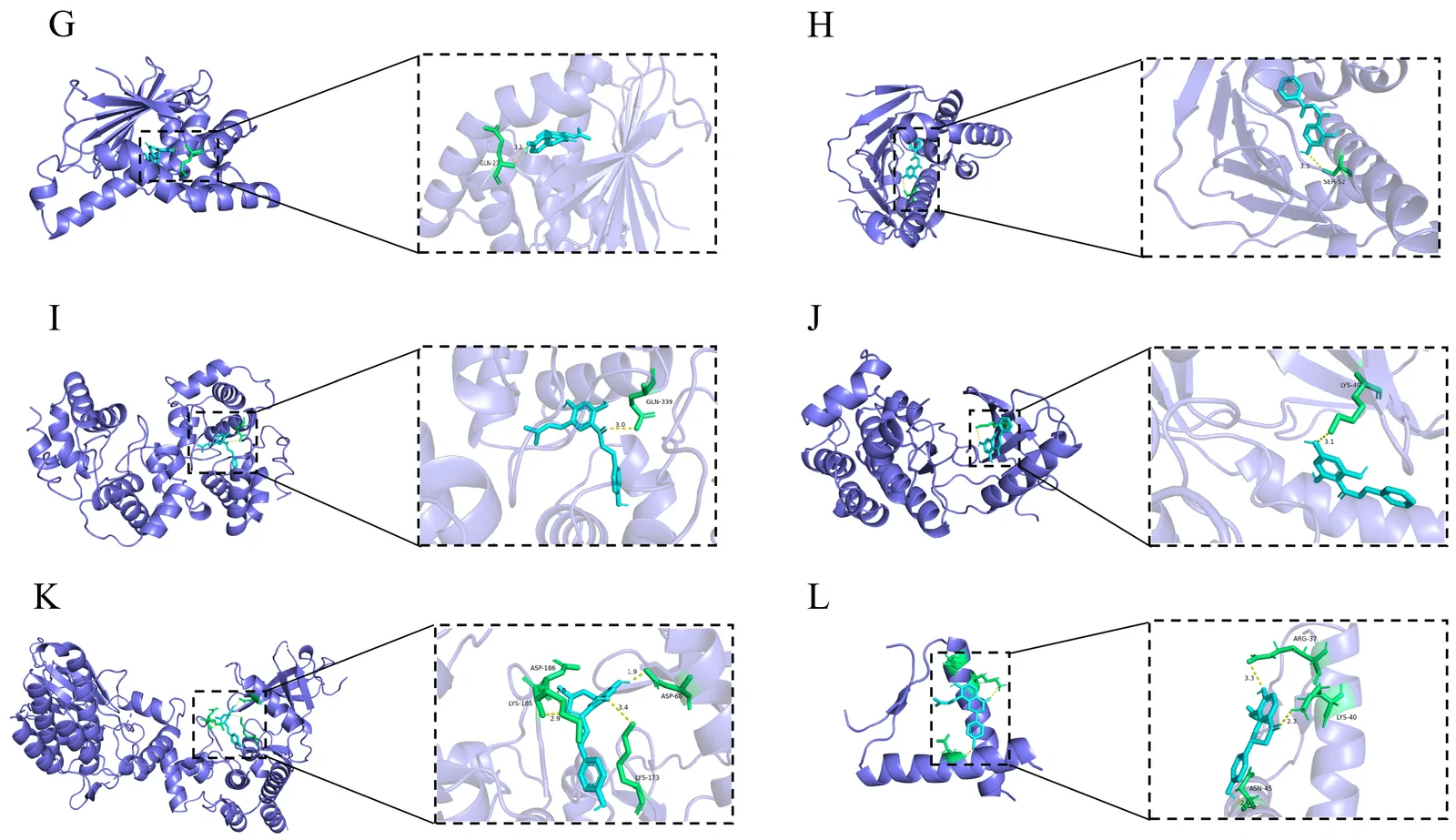
Network pharmacology and molecular docking results. (**A**) Target map of component and disease intersection. (**B**) Map of core targets. (**C**) Diagram of the component-target network. (**D**) GO enrichment analysis graph. (**E**) KEGG pathway enrichment analysis map. (**F**) Molecular docking thermal energy map. (**G**) *HSP90AA1*-Bavachinin. (**H**) *HSP90AA1*-Chrysin. (**I**) *AKT1*-Bavachalcone. (**J**) *EGFR*-Chrysin. (**K**) *STAT3*-Bavachalcone. (**L**) *TP53*-Isobavachin.

A total of 3033 related component targets were obtained from SwissTargetPrediction and PharmaMapper databases, and 3653 hyperlipemia-related targets were obtained from genecard and omim databases. A total of 215 common targets were obtained via intersection analysis (Figure 6A).

#### 3.4.2. PPI Network Analysis

In this study, the 215 overlapping targets were submitted to the STRING database to construct a protein–protein interaction (PPI) network. The resulting PPI network contained 213 nodes and 3455 edges, with an average node degree of 32.4, an average local clustering coefficient of 0.561, and a PPI enrichment *p*-value < 1.0 × 10^−16^ (Figure 6B). Subsequently, the PPI data were imported into Cytoscape for visual analysis and core target screening. Furthermore, the overlapping genes were visualized in Cytoscape to generate the compound–target network (Figure 6C).

The top five core targets were screened by topological analysis, including *TP53*, *STAT3*, *AKT1*, *HSP90AA1* and *EGFR*. These targets may play central regulatory roles in the onset, progression, and intervention of hyperlipidemia. *TP53* is involved in the regulation of cell cycle, DNA repair and apoptosis, and its dysfunction is closely related to tumor and inflammation [97]. In this study, *TP53* is located at the core of the PPI network, suggesting that polyphenols from SLKDT may exert lipid-lowering effects by regulating *TP53* and its downstream pathways. Previous studies had confirmed that the activation of *p53* could significantly down-regulate the rate-limiting enzyme *CYP7A1* in the bile acid synthesis pathway and reduce the production of bile acids, which leads to the blocking of cholesterol decomposition and abnormal accumulation of intracellular lipids [98]. *STAT3* is the core component of the *JAK/STAT* pathway. This protein participates in cell proliferation, immune responses and inflammatory reactions, and mediates tumor survival, immune evasion and inflammatory injury [99]. In the present study, *STAT3* exhibited strong binding interactions with *TNF*, *IL2*, *AKT1* and *MAPK1*, serving as a critical hub linking inflammatory processes and cell proliferation. Accumulating evidence has demonstrated that inhibiting *STAT3* expression and phosphorylation blocks the downstream *SREBP-1*-mediated lipogenic pathway. This process suppresses the key lipogenic enzymes *ACC1* and *FAS*, thereby attenuating de novo hepatic lipogenesis and lipid accumulation [100]. As a central mediator of the *PI3K-Akt* signaling pathway, *AKT1* governs cellular metabolism, survival and apoptosis [101]. It also facilitates adipogenic differentiation via the modulation of lipin-1 expression [102]. Its intermolecular interactions with *TP53* and *STAT3* indicate that lipid metabolism regulation can coordinately modulate cell viability and inflammatory signaling. Furthermore, other high-priority targets including *TNF*, *HSP90AA1* and *EGFR* separately regulate inflammatory signal transduction, the folding stability of signaling proteins and cell proliferation. Collectively, these targets form a multi-target, multi-pathway molecular regulatory network.

#### 3.4.3. Compound–Target Construction

Compound–target network analysis uncovered interactive associations between SLKDT-derived polyphenolic components and 215 overlapping therapeutic targets (Figure 6C). Further network screening identified five core bioactive compounds, including chrysin, 6-demethoxytangeretin, bavachinin, isobavachin, and bavachalcone, all of which belong to the flavonoid family. These flavonoid ingredients can alleviate metabolic inflammation and oxidative stress, which is primarily responsible for the hypolipidemic capacity of SLKDT polyphenols [103].

#### 3.4.4. Enrichment Analysis of GO and KEGG Pathways

The Metascape database was used to analyze the functions and pathways of the potential genes of polyphenols in SLKDT. The results of GO enrichment analysis are shown in Figure 6D. In terms of lipid metabolism, GO analysis identified 816 biological processes (BP), 97 cellular components (CC), and 226 molecular functions (MF). The top 30 GO terms were selected from the analysis results, including 10 biological processes (BP), 10 cellular components (CC) and 10 molecular functions (MF). Regarding biological processes (BP), the cellular response to amyloid-beta exhibited the highest enrichment score. Meanwhile, the epidermal growth factor receptor signaling pathway, *PI3K/Akt* signaling pathway, positive regulation of the *MAPK* cascade, and *ERK1/ERK2* cascade regulation were also significantly enriched as key functional pathways. Overall, these enriched functional items constituted a sophisticated regulatory network centered on lipid metabolism processes, hubbed by the *PI3K/Akt* and *MAPK/ERK* signaling pathways. This network is closely implicated in the pathological progression of lipid metabolic disorders under hyperlipidemic conditions.

At the CC level, the items with the highest enrichment scores were membrane raft and receptor complex. Other highly enriched components included the perinuclear region of the cytoplasm, protein-containing complex, cell surface, extracellular exosome, mitochondrion, cytosol, plasma membrane, and cytoplasm. These findings indicated that the target proteins were predominantly distributed in cell membrane signaling microdomains (membrane raft and plasma membrane), mitochondria, and extracellular vesicles. Functionally, these proteins are primarily involved in signal reception, lipid transport, metabolic processes, and metabolite transport, thereby participating in the regulation of lipid metabolism.

At the MF level, protein tyrosine kinase activity exhibited the highest enrichment score. The secondary significantly enriched terms primarily comprised protein kinase activity, enzyme binding, protein serine kinase activity, and protein serine/threonine kinase activity. In addition, identical protein binding, ATP binding, hydrolase activity, and nucleotide binding were also highly enriched. These results demonstrated that the core molecular functions were dominated by kinase activity (tyrosine/serine/threonine kinase), enzyme binding, and nucleotide binding. These functional characteristics indicate that the target genes play pivotal roles in mediating protein phosphorylation, modulating the activity of lipid metabolism-related enzymes, and facilitating the binding of signaling molecules. In this study, the lipid metabolic process was significantly enriched, indicating that the target genes were involved in the regulation of metabolic processes such as lipid synthesis, decomposition, and transport under hyperlipemia. Moreover, the *PI3K/Akt* and *MAPK/ERK* signaling pathways were enriched in biological processes. The *PI3K/Akt* pathway can activate downstream *mTOR* and *SREBP1/2* transcription factors, thereby directly modulating the expression of key lipid-metabolizing enzymes, including fatty acid synthase (*FASN*) and *HMG-CoA* reductase, to facilitate lipid synthesis and accumulation [104]. The *MAPK/ERK* pathway modulates nuclear receptors including peroxisome proliferator-activated receptors (*PPARs*) and liver X receptor (*LXR*) via phosphorylation, thereby regulating lipid oxidation and transport processes [105]. Cellular component enrichment results demonstrated that membrane rafts serve as enrichment sites for *EGFR* and core *PI3K/Akt* pathway molecules, supporting the assembly and signal transduction of these signaling cascades. Mitochondrion enrichment suggested that target genes participated in fatty acid β-oxidation and modulate lipid catabolism. In hyperlipidemia, mitochondrial dysfunction suppresses lipid degradation and exacerbates lipid accumulation [106]. Furthermore, the enrichment of extracellular exosomes indicated that these genes mediated intercellular transmission of lipid transport and metabolic signals via exosomes, thereby participating in the regulation of systemic lipid homeostasis [107]. Molecular functional enrichment analysis revealed prominent enrichment of protein tyrosine kinase activity, which further explains the regulatory mechanism of lipid metabolism at the molecular level. As upstream signaling receptors, tyrosine kinases trigger downstream lipid metabolic programs by activating the *PI3K/Akt* and *MAPK/ERK* pathways [108]. In contrast, serine/threonine kinases directly phosphorylate key lipid metabolic enzymes, including *ACC* and *HMGCR* [109].

In this study, a total of 178 signaling pathways were obtained via KEGG functional analysis, and the top 20 pathways were visualized (Figure 6E). KEGG enrichment analysis identified significant enrichment of the lipid and atherosclerosis, *HIF-1*, *PI3K-Akt*, *MAPK*, *AMPK*, *TNF*, and *NF-κB* signaling pathways. These results indicate that SLKDT polyphenols exert lipid-regulating effects through the synergistic modulation of multiple signaling pathways. Among these pathways, the lipid and atherosclerosis pathway shows the most significant enrichment. As the core pathological pathway for hyperlipidemia and its complications, indicating that SLKDT polyphenols directly target key pathological processes such as lipid deposition and vascular endothelial injury to retard the progression of hyperlipidemia into atherosclerosis [110]. The *PI3K-Akt* and *MAPK* signaling pathways modulate downstream transcription factors, including *SREBP* and *PPARs*. They also regulate the activity of key metabolic enzymes such as fatty acid synthase and *HMG-CoA* reductase. Through these regulatory effects, the two pathways suppress lipid synthesis, facilitate lipid oxidation and transport, and ultimately ameliorate lipid metabolism disorders [111,112,113]. The *AMPK* signaling pathway is central to the development of lipid metabolic disorders. *EGFR*, an upstream membrane receptor, initiates the *PI3K-AKT1* signaling cascade following its activation, which in turn inhibits *AMPK* activity. Reduced *AMPK* function over-activates the *SREBP* pathway, ultimately driving excessive biosynthesis of fatty acids and cholesterol [36]. Furthermore, the enrichment of the *AMPK* signaling pathway further confirmed its role in promoting fatty acid β-oxidation and suppressing lipid synthesis. Meanwhile, the enrichment of the insulin resistance pathway suggested that SLKDT polyphenols improved the glucose and lipid metabolism imbalance caused by hyperlipidemia. In addition, enrichment of inflammatory pathways including *TNF* and *NF-κB* was identified in this analysis. This finding revealed that SLKDT polyphenols suppressed chronic low-grade inflammation. The polyphenols disrupted the vicious cycle consisting of lipid accumulation, inflammatory activation and metabolic disturbance, and relieved tissue damage associated with hyperlipidemia. In conclusion, SLKDT polyphenols modulated the network of lipid metabolism, signal transduction and inflammatory responses via a multi-component, multi-target and multi-pathway mode. This observation laid a scientific foundation for explaining their anti-hyperlipidemia mechanism and offered guidance for further mechanistic validation.

#### 3.4.5. Molecular Docking

To clarify the potential mechanism through which the core active ingredients regulated lipid metabolism, we employed molecular docking technology to investigate the interaction between the core polyphenol components in SLKDT and target proteins. The results are presented in Figure 6F–L. Our research findings demonstrated that each active ingredient could stably bind to its corresponding target protein and form stable complexes with key amino acid residues through non-covalent interactions, including hydrogen bonds. As examples, bavachinin formed hydrogen bonds with the GLN-23 residue of *HSP90AA1*, chrysin interacted with the SER-52 residue of *HSP90AA1*, and bavachalcone bound to the GLN-339 residue of *AKT1*. The binding distances were within a reasonable range, suggesting that the active ingredient had a good binding affinity with the target protein. The binding heatmap indicated that the binding free energy of most constituents to target proteins was lower than −6.0 kcal/mol. Multiple components produced binding energies below −8.5 kcal/mol when docked with proteins *AKT1* and *HSP90AA1*. Several compound–protein pairs reached binding free energies as low as −9.9 kcal/mol, which reflected strong high-affinity interactions. Furthermore, molecular docking predicted that multiple active constituents of SLKDT polyphenols could bind to key hyperlipidemia target proteins with high affinity, which may affect their conformation and function. Among these target proteins, *HSP90AA1* was simultaneously targeted by multiple components, which served as the core target responsible for the anti-hyperlipidemia effect of SLKDT polyphenols. Among the identified compounds, chrysin, bavachinin, and isobavachin exhibited strong binding capacities toward multiple targets, which could serve as the predominant core active substances of SLKDT. Chrysin promoted fatty acid oxidation and suppressed fatty acid synthesis by downregulating *SREBP1*, *FAS*, and *ACC1*, which was highly consistent with our in vitro experimental results. Furthermore, chrysin modulated glucose and lipid metabolism through the *AMPK/PI3K/AKT* signaling pathway, a conclusion further corroborated by KEGG pathway enrichment analysis [114]. Molecular docking results demonstrated that the active constituents of SLKDT polyphenols directly modulated the conformation and functional activity of key hyperlipidemia-related target proteins via specific binding. Through such interactions, these compounds interfered with signaling pathways involved in lipid metabolism, inflammatory responses, and hypoxic stress, thereby exerting lipid-regulating effects.

However, since network pharmacology results are primarily based on database predictions, only the downstream genes of the core target proteins identified in this study were validated by cellular RT-qPCR, whereas the core target proteins themselves have not yet been verified by RT-qPCR or Western blotting. Future studies will employ these molecular biological approaches to validate their mRNA and protein expression levels, thereby further confirming the molecular mechanism underlying the lipid-lowering effect.

## 4. Conclusions

In conclusion, this study optimized and established an ultrasonic extraction process for polyphenols from SLKDT, achieving a yield of up to 131.88 mg/g. In vitro cellular experiments confirmed the hypolipidemic activity of SLKDT polyphenols and clarified their regulatory effects on lipid metabolism-related genes such as *ACC*, *HMGCR*, *SREBP-1c*, and *FASN*. Furthermore, using LC-MS/MS, network pharmacology, and molecular docking, the lipid-lowering active components in SLKDT polyphenols were preliminarily screened, with chrysin, 6-demethoxytangeretin, bavachinin, isobavachin, and bavachalcone as key compounds. These components regulate lipid metabolism by targeting core proteins such as *TP53*, *STAT3*, and *AKT1*, and synergistically modulating pathways including atherosclerosis, *PI3K-Akt*, and *AMPK*. This study systematically established an efficient ultrasonic extraction process for polyphenols from SLKDT and clarified their lipid-lowering activity and underlying molecular mechanisms, as well as the core active components and key target signaling pathways. These findings fill the research gap regarding the material basis and molecular mechanism of SLKDT polyphenols in regulating lipid metabolism, and provide solid experimental evidence and theoretical support for the development and application of SLKDT as a natural lipid-lowering functional raw material. Nevertheless, this study has certain limitations. The lipid-lowering effects were only verified using in vitro cell models, while in vivo animal experiments were not performed. In addition, the independent efficacy of each core active ingredient and their synergistic mechanisms have not been fully explored, and the detailed regulatory patterns of the involved signaling pathways remain to be further elucidated. In future studies, hyperlipidemia animal models will be constructed to validate the in vivo lipid-lowering efficacy and metabolic characteristics of SLKDT polyphenols, so as to supplement and improve the pharmacodynamic evidence. Furthermore, the core active compounds identified in this study, such as chrysin and 6-demethoxytangeretin, will be further isolated and purified. The individual lipid-lowering effects of each single component will be investigated, and multiple biological techniques will be combined to clarify the molecular mechanisms by which these components synergistically regulate lipid metabolism and alleviate vascular lipid deposition.

## Figures and Tables

**Table 1:** Factors and levels of polyphenol extraction from SLKDT.

Levels	Factors			
	A. Ethanol Volume Fraction (%)	B. Ultrasound Time (min)	C. Solid-to-Liquid Ratio (g/mL)	D. Ultrasonic Temperature (°C)
−1	30	30	40	45
0	40	40	50	55
1	50	50	60	65

**Table 2:** The primer sequences of related genes.

Gene	Forward Primer (5′–3′)	Reverse Primer (5′–3′)
*TBP*	GTTCAGCAGTCAACGTCCCAG	GTCATGGGGGAGGGATACAGTG
*ACC*	GTTATGTGAAAGATGTGGATGA	TGTCTGAAGAGATTAGGGAAGT
*FASN*	GCTTCCGAGATTCCATCCTACG	GCAGTCAGGCTCACAAACGA
*HMGCR*	CTTGTGTGTCCTTGGTATTAGAGCTT	GCTGAGCTGCCAAATTGGA
*SREBP-1c*	CCACTTCATCAAGGCAGACTCG	CAAGATGGTTCCGCCACTCAC
*CYP7A1*	TTACAAGGCGGGACACACAG	CCTCAAGCTCTCTGCCAGTT

**Table 3:** Experimental design of response surface methodology.

Number	Factors				
	A: Ethanol Volume Fraction (%)	B: Ultrasound Time (min)	C: Solid-to-Liquid Ratio (g/mL)	D: Ultrasonic Temperature (°C)	Polyphenol Yield (mg/g)
1	50	40	65	50	131.4
2	40	40	55	50	131.75
3	40	50	55	60	122.5
4	40	40	55	50	132
5	50	40	45	50	121.8
6	50	50	55	50	128.5
7	40	40	55	50	130.75
8	40	40	65	60	125.4
9	40	30	65	50	127.5
10	40	40	55	50	129.75
11	40	30	55	40	121
12	40	40	45	60	118.2
13	50	40	55	60	123
14	40	50	45	50	120
15	40	30	55	60	121.5
16	40	40	45	40	118
17	30	30	55	50	121.5
18	50	40	55	40	124.5
19	40	50	65	50	129.6
20	30	50	55	50	123.75
21	30	40	55	60	120.5
22	40	30	45	50	117.8
23	50	30	55	50	125.5
24	40	40	65	40	129.9
25	30	40	65	50	126.9
26	30	40	55	40	121
27	40	40	55	50	131
28	30	40	45	50	118.4
29	40	50	55	40	124.25

**Table 4:** Analysis of variance of experimental results.

Sources of Variance	Sum of Squares	Degree of Freedom	Mean Square	F-Value	*p*-Value	Significance
Model	599.49	14	42.82	68.57	<0.0001	**
A. Ethanol volume fraction	15.87	1	15.87	25.41	0.0002	**
B. Ultrasound time	42.75	1	42.75	68.46	<0.0001	**
C. Solid-to-liquid ratio	4.75	1	4.75	7.61	0.0154	*
D. Ultrasonic temperature	266.02	1	266.02	426.01	<0.0001	**
AB	0.1406	1	0.1406	0.2252	0.6424	
AC	1.27	1	1.27	2.03	0.1765	
AD	0.0025	1	0.0025	0.0040	0.9504	
BC	0.2500	1	0.2500	0.4004	0.5371	
BD	0.3025	1	0.3025	0.4844	0.4978	
CD	5.52	1	5.52	8.84	0.0101	*
A^2^	80.98	1	80.98	129.68	<0.0001	**
B^2^	62.92	1	62.92	100.77	<0.0001	**
C^2^	178.08	1	178.08	285.17	<0.0001	**
D^2^	72.61	1	72.61	116.28	<0.0001	**
Residual error	8.74	14	0.6244			
Misfitting item	5.57	10	0.5567	0.7014	0.7051	Not significant
Pure error	3.17	4	0.7937			
Total error	608.23	28				

* *p* < 0.05, ** *p* < 0.01.

**Table 5:** LC-MS/MS analysis of polyphenols in SLKDT.

Number	Name	Molecular Formula	RT (min)	Class	POS Q1	POS Q3	NEG Q1	NEG Q3	Peak Area
1	Galangin-7-O-glucoside	C_21_H_20_O_10_	10.69	Glycosides	433.111	271.06			218,413,260.10 ± 6,288,083.23
2	Apigenin-7-O-neohesperidoside	C_27_H_30_O_14_	11.11	Glycosides	579.17	271.07	577.16	269.05	184,912,260.77 ± 583,901.07
3	Kaempferol-3-O-glucoside	C_21_H_20_O_11_	10.56	Glycosides	449.11	287.062			180,369,410.50 ± 8,198,167.31
4	Luteolin-7-O-glucoside	C_21_H_20_O_11_	10.57	Glycosides	449.11	287.2	447.1	285.04	161,302,181.60 ± 6,408,722.46
5	Kaempferol-3-O-rutinoside	C_27_H_30_O_15_	10.4	Glycosides	595.2	287.09	593.15	285.04	146,199,944.73 ± 3,807,925.71
6	Luteolin-7-O-rutinoside	C_27_H_30_O_15_	10.42	Glycosides	595.17	449.102			130,513,944.00 ± 3,471,830.83
7	Kaempferol-3-O-(6″-galloyl) galactoside	C_28_H_24_O_15_	10.42	Glycosides	601.12	287.06			102,390,267.26 ± 4,905,540.89
8	Quercetin-7-O-glucoside	C_21_H_20_O_12_	10.31	Glycosides			463.09	300.7	68,838,218.86 ± 8,553,669.99
9	Hesperetin-5-O-glucoside	C_22_H_24_O_11_	10.31	Glycosides			463.12	301	60,899,542.10 ± 1,897,365.01
10	Luteolin-7-O-neohesperidoside	C_27_H_30_O_15_	10.42	Glycosides	595.17	449.102			50,210,518.70 ± 332,940.16
11	Eriodictyol-7-O-glucoside	C_21_H_22_O_11_	10.56	Glycosides	451.12	289			45,363,214.72 ± 1,816,846.55
12	Luteolin-7-O-gentiobioside	C_27_H_30_O_16_	8.62	Glycosides	611.16	287.052			42,889,831.82 ± 3,280,662.56
13	Luteolin-7,3′-di-O-glucoside	C_27_H_30_O_16_	8.86	Glycosides	611.16	287.052			40,592,307.97 ± 3,142,217.40
14	Kaempferol-3,7-di-O-glucoside	C_27_H_30_O_16_	8.86	Glycosides	611.16	287.06			28,045,200.04 ± 2,155,038.64
15	Isorhamnetin-3-O-Glucoside	C_22_H_22_O_12_	10.69	Glycosides	479.12	317.062	477.11	314.04	7,879,920.36 ± 103,987.12
16	Isorhamnetin-7-O-glucoside	C_22_H_22_O_12_	10.69	Glycosides	479.12	317.062			6,727,534.99 ± 756,935.80
17	Kaempferol-3-O-arabinoside	C_20_H_18_O_10_	10.64	Glycosides	419.1	287.05			5,391,047.07 ± 409,656.91
18	Quercetin-3-O-sophoroside	C_27_H_30_O_17_	9.63	Glycosides	627.16	303.04			3,741,973.37 ± 1,461,727.11
19	Orientin-2″-O-galactoside	C_27_H_30_O_16_	9.88	Glycosides	611.16	449.11			2,635,034.31 ± 263,976.68
20	Acteoside	C_29_H_36_O_15_	11.45	Glycosides	625.2	565.18	623.2	461.17	2,358,275.80 ± 155,482.22
21	Quercetin-3-O-sophoroside-7-O-rhamnoside	C_33_H_40_O_21_	9.17	Glycosides	773.21	303.05			2,018,275.89 ± 228,360.13
22	Syringaldehyde-4-O-glucoside	C_15_H_20_O_9_	8.63	Glycosides			343.11	181.05	1,602,325.18 ± 154,455.72
23	Acacetin-7-O-glucoside	C_22_H_22_O_10_	10.84	Glycosides	447.13	285.18	445.12	283.06	1,246,991.22 ± 514,839.57
24	Quercetin-3-O-(2″-O-galactosyl)glucoside	C_27_H_30_O_17_	8.57	Glycosides	627.16	465.1			1,175,453.70 ± 53,399.23
25	Apigenin-4′-O-rhamnoside	C_21_H_20_O_9_	9.44	Glycosides			415.1	269.05	1,054,469.58 ± 15,929.48
26	Kaempferol-3-O-robinobioside	C_27_H_30_O_15_	10.57	Glycosides			593.15	285	767,618.80 ± 1,263,577.43
27	3-Hydroxy-5-Methylphenol-1-O-Glucoside	C_13_H_18_O_7_	7.75	Glycosides			285.1	123	547,403.70 ± 98,940.77
28	Pinocembrin-7-O-glucoside	C_21_H_22_O_9_	10.05	Glycosides	419.132	257.08			368,882.37 ± 61,756.00
29	Luteolin-4′-O-glucoside	C_21_H_20_O_11_	8.24	Glycosides	449.11	287.05			279,671.34 ± 48,413.49
30	Quercetin-3-O-glucoside	C_21_H_20_O_12_	7.97	Glycosides	465.11	285.04	463.09	300.03	259,374.31 ± 72,688.52
31	Rutin	C_27_H_30_O_16_	10.82	Flavonoids	611.2	303.04	609.15	301	109,689,776.07 ± 3,320,024.46
32	Isohyperoside	C_21_H_20_O_12_	10.33	Flavonoids	465.1	303.06			75,844,735.88 ± 2,669,409.67
33	Bavachinin	C_21_H_22_O_4_	9.03	Flavonoids	339.16	147.04	337.1	119.05	73,634,077.54 ± 6,952,358.34
34	Apigenin	C_15_H_10_O_5_	10.98	Flavonoids	271.06	153.01	269.051	151	25,554,800.63 ± 1,267,096.44
35	2′-Hydroxygenistein	C_15_H_10_O_6_	10.78	Flavonoids	287.06	153.02			23,122,694.71 ± 945,407.30
36	Isoscutellarein	C_15_H_10_O_6_	10.99	Flavonoids	287.06	153.01			7,448,779.40 ± 199,775.75
37	Amentoflavone	C_30_H_18_O_10_	9.46	Flavonoids			537.08	375.05	6,791,618.41 ± 2,637,979.81
38	Isosakuranetin	C_16_H_14_O_5_	9.97	Flavonoids	287.09	153.02			6,560,153.64 ± 1,402,722.19
39	6-Demethoxytangeretin	C_19_H_18_O_6_	8.66	Flavonoids			341.101	206.06	2,883,413.96 ± 413,592.54
40	Nepetin	C_16_H_12_O_7_	7.58	Flavonoids	317.07	302.04	315.051	151	2,330,238.94 ± 217,388.43
41	Epicatechin	C_15_H_14_O_6_	1.66	Flavonoids	291.1	139.04			1,433,349.76 ± 119,246.93
42	Tiliroside	C_30_H_26_O_13_	10.57	Flavonoids	595.1	147.04	593.131	285.04	1,091,007.25 ± 161,541.72
43	Isobavachin	C_20_H_20_O_4_	8.85	Flavonoids			323.13	119.05	965,293.82 ± 136,866.30
44	Didymin	C_28_H_34_O_14_	10.57	Flavonoids	595.2	287.09	593.19	285.08	915,891.17 ± 80,432.84
45	Bavachalcone	C_20_H_20_O_4_	8.78	Flavonoids			323.131	119.05	905,856.33 ± 102,306.61
46	Diosmetin-7-O-rutinoside	C_28_H_32_O_15_	10.64	Flavonoids	609.18	301	607.171	299.06	874,101.32 ± 56,365.15
47	Nicotiflorin	C_27_H_30_O_15_	10.57	Flavonoids	595.171	287.06	593.15	285.04	746,246.65 ± 71,405.87
48	6,7,8-Tetrahydroxy-5-methoxyflavone	C_16_H_12_O_6_	11.02	Flavonoids	301.07	286.051			436,965.29 ± 21,269.88
49	Hispidulin	C_16_H_12_O_6_	11.02	Flavonoids	301.07	286.052			434,907.66 ± 20,480.31
50	Nepetin	C_16_H_12_O_7_	11.42	Flavonoids	317.07	302.04	315.051	151	424,208.42 ± 102,484.43
51	Noreugenin	C_10_H_8_O_4_	8.19	Flavonoids			191.032	102.95	392,314.97 ± 66,263.52
52	Calycosin-7-O-glucoside	C_22_H_22_O_10_	10.15	Flavonoids	447.1	285.08			385,480.29 ± 169,090.05
53	Loureirin A	C_17_H_18_O_4_	9.96	Flavonoids			285.11	134.04	368,854.58 ± 129,568.41
54	Methylnissolin	C_17_H_16_O_5_	7.77	Flavonoids			299.1	179.03	319,969.02 ± 54,609.66
55	Apigenin 7-O-beta-D-glucuronide	C_21_H_18_O_11_	10.92	Flavonoids	447.1	271.06	445.08	269.05	312,735.83 ± 44,618.25
56	Sakuranetin	C_16_H_14_O_5_	9.97	Flavonoids			285.08	165.02	274,564.79 ± 79,926.71
57	Chrysin	C_15_H_10_O_4_	10.89	Flavonoids	255.07	153	253.05	209.06	272,340.86 ± 42,346.21
58	Isorhamnetin	C_16_H_12_O_7_	7.81	Flavonoids	317.07	285.04	315.05	151	243,703.50 ± 69,905.92
59	Genistein	C_15_H_10_O_5_	8.24	Flavonoids			269.05	225.05	238,297.16 ± 39,036.63
60	Ononin	C_22_H_22_O_9_	10.68	Flavonoids	431.1	269.1	429.12	195.07	229,465.40 ± 43,029.08
61	Hydroxytyrosol	C_8_H_10_O_3_	7.71	Phenols			153.05	123.04	44,069,199.18 ± 2,789,639.14
62	4-Hydroxybenzoic acid	C_7_H_6_O_3_	9.02	Phenols			137.02	93	30,906,192.89 ± 954,890.14
63	1-O-Salicyl-D-glucose	C_13_H_16_O_8_	8.55	Phenols			299.08	137.021	12,200,618.51 ± 1,856,982.55
64	6-O-Caffeoyl-D-glucose	C_15_H_18_O_9_	1.46	Phenols			341.09	179.03	11,026,233.05 ± 1,379,462.40
65	Coniferin	C_16_H_22_O_8_	1.52	Phenols			341.12	179	8,953,301.90 ± 1,630,959.83
66	4-O-Glucosyl-3,4-dihydroxybenzyl alcohol	C_13_H_18_O_8_	7.73	Phenols			301.09	139.05	5,551,294.53 ± 921,959.45
67	[8]-Shogaol	C_19_H_28_O_3_	9.76	Phenols	305.21	137.06			2,055,739.38 ± 367,303.30
68	3,4-Dimethoxyphenol	C_8_H_10_O_3_	5.94	Phenols			153.05	111.04	1,399,193.67 ± 254,753.70
69	4-Hydroxy-3-methoxymandelate	C_9_H_10_O_5_	8.61	Phenols			197	153	617,608.05 ± 70,310.54
70	4-Allylcatechol	C_9_H_10_O_2_	7.98	Phenols			149.06	121.03	499,181.33 ± 146,521.41
71	4-O-(6′-O-Glucosylcaffeoylglucosyl)-4-hydroxybenzyl alcohol	C_28_H_34_O_15_	9.26	Phenols			609.18	447.13	434,730.00 ± 42,902.03
72	2′,4′,6′-Trihydroxyacetophenone	C_8_H_8_O_4_	6.94	Phenols			167.03	123.05	259,340.88 ± 61,323.03
73	(E)-m-Coumaric acid	C_9_H_8_O_3_	9.9	Phenolic acids			163.041	119.051	34,272,173.79 ± 1,545,452.97
74	Protocatechuic acid	C_7_H_6_O_4_	8.2	Phenolic acids			153.02	109.032	26,767,646.47 ± 3,110,759.76
75	Caffeic acid	C_9_H_8_O_4_	9.35	Phenolic acids			179.03	135.05	22,839,250.23 ± 720,683.95
76	2,5-Dihydroxybenzoic acid	C_7_H_6_O_4_	8.04	Phenolic acids			153.02	109.031	20,275,977.18 ± 17,399,486.00
77	Homovanillic acid	C_9_H_10_O_4_	7.77	Phenolic acids			181.2	137.2	6,635,367.18 ± 1,948,018.76
78	p-Hydroxyphenylacetic acid	C_8_H_8_O_3_	6.39	Phenolic acids	153.05	109.03	151.04	107.001	489,169.50 ± 210,306.36
79	Syringic acid	C_9_H_10_O_5_	7.95	Phenolic acids			197.05	123	480,538.94 ± 68,055.41
80	Vanillic acid	C_8_H_8_O_4_	6.72	Phenolic acids			167.03	108.02	394,376.49 ± 100,241.34
81	Chlorogenic acid	C_16_H_18_O_9_	9.84	Phenolic acids			353.09	191.01	362,247.00 ± 41,238.46
82	Rosmarinic acid	C_18_H_16_O_8_	1.2	Phenolic acids			359.1	161.02	358,563.23 ± 58,404.85
83	3,4-Dihydroxybenzeneacetic acid	C_8_H_8_O_4_	6.94	Phenolic acids			167.03	123.04	323,236.15 ± 149,690.53
84	Gallic acid	C_7_H_6_O_5_	6.77	Phenolic acids			169.01	125	260,158.76 ± 31,310.55
85	p-Coumaric acid	C_9_H_8_O_3_	9.13	Phenolic acids	165.05	119	163.042	119.05	8,801,497.39 ± 1,718,703.99
86	Vanilloloside	C_14_H_20_O_8_	7.9	Others			315.11	153.02	62,415,769.32 ± 9,967,807.12
87	Piceatannol	C_14_H_12_O_4_	1.26	Others	245.08	199.08	243.07	159.1	5,158,760.98 ± 1,372,764.43
88	Plantagoside	C_21_H_22_O_12_	6.19	Others			465.1	303.05	2,148,500.97 ± 3224.28

Note: Q1 is set for the m/z of the precursor ion, while Q3 corresponds to the m/z of the product ion.

## Data Availability

The raw data supporting the conclusions of this article will be made available by the authors on request.

## Supplementary Materials

The following supporting information can be downloaded at: https://www.mdpi.com/article/10.3390/foods15203667/s1, Figure S1: cell viability.
